# Comparative Chemical Analysis of Eight *Punica granatum* L. Peel Cultivars and Their Antioxidant and Anti-Inflammatory Activities

**DOI:** 10.3390/antiox11112262

**Published:** 2022-11-16

**Authors:** Valentina Parisi, Valentina Santoro, Giuliana Donadio, Maria Laura Bellone, Gianfranco Diretto, Carla Sandri, Francesca Mensitieri, Nunziatina De Tommasi, Fabrizio Dal Piaz, Alessandra Braca

**Affiliations:** 1 Department of Pharmacy, University of Salerno, 84084 Fisciano, SA, Italy; 2 PhD Program in Drug Discovery and Development, Department of Pharmacy, Università degli Studi di Salerno, Via Giovanni Paolo II 132, 84084 Fisciano, SA, Italy; 3 Casaccia Research Centre, Biotechnology Laboratory, Italian National Agency for New Technologies, Energy and Sustainable Development (ENEA), 00123 Rome, Italy; 4 Dipartimento di Medicina, Chirurgia e Odontoiatria “Scuola Medica Salernitana”, Università degli Studi di Salerno, via Giovanni Paolo II 132, 84084 Fisciano, SA, Italy; 5 Department of Pharmacy, University of Pisa, 56126 Pisa, Italy

**Keywords:** *Punica granatum*, pomegranate, mass spectrometry, phenolic compounds, antioxidant, anti-inflammatory, bioinformatics analyses

## Abstract

A comparative quali-quantitative study of the peel extracts of eight *Punica granatum* cultivars obtained from underexploited areas of South Italy was carried out in order to valorize them as health-promoting by-products. The results showed that all of the samples possessed 45 ellagitannins, consisting mainly of polyhydroxyphenoyls; 10 flavonoids, belonging to flavonol, flavone, and catechin classes; and 2 anthocyanins. The most representative compounds underwent quantification through a LC-MS/MS multiple reaction monitoring (MRM)-based method; their qualitative profile was almost superimposable, while variability in the quantitative phenolic content was observed. The antioxidant activity was investigated using cell-free and cell-based assays. The in vitro anti-inflammatory potential was also studied by monitoring three typical markers of inflammation (IL-1β, IL-6, and TNF-α). Moderate differences in both activities were observed between the cultivars. Results showed that all of the investigated peels have a potential use as healthy bioactive phytocomplexes due to the interesting antioxidant and anti-inflammatory activities; in particular from the bioinformatic approaches a series of compounds, including galloyl-, pedunculagin- and ellagic acid-based, were found to be highly correlated with bioactivity of the extracts. Finally, the bioactivities showed by a Campanian local cultivar, ‘Granato di Aiello del Sabato’, could promote its cultivation by local farmers and germplasm conservation.

## 1. Introduction

*Punica granatum* L. (Lythraceae), commonly known as pomegranate, is a small tree native to Iran, China, and India and is widely cultivated in the Mediterranean region, North and South Africa, Asia, and Central and South America due to its commercial attractiveness as a fresh fruit or as juice. It’s a temperate climate species capable of easily spreading in arid and semi-arid areas and is tolerant to salinity and water deficiency and agronomic factors that usually reduce the growth of other crops. The pomegranates’ successful adaptation to Mediterranean weather conditions has led to their diffusion in this area and the propagation of new varieties. Since ancient times, the usage of pomegranate has been reported in many prehistoric human cultures [1], while recently it has been described as a “super food” and classified among the top ten fruits with high nutraceutical value. The juice, mostly marketed instead of fresh fruit to avoid the unpleasant removal of the seeds, is a rich source of polyphenols such as anthocyanins (glycosides of cyanidin, delphinidin, and pelargonidin), flavonoids (proanthocyanidins and flavanols), and tannins (ellagitannins and gallotannins) [2] as well as volatile substances with an intense aroma [3]. The phenolic content of pomegranate juice varies among the different cultivars, varieties, and genotypes (actually more than 500), climates, agronomical conditions, harvest and post-harvest times, juice obtaining method, and its processing, leading to different health-promoting products widely used not only as food but also in nutraceutical and cosmetic preparations [4].

The by-product of *P. granatum* is estimated to be composed of 80% peels and 20% seeds; therefore, in this context, the impact of this bio-waste is mainly addressed to discharge, representing a significant issue for the agro-food industry and requiring strategic action for the agricultural production and processing industry. Recently, the increasing attention to avoid environmental pollution, as well as to the rationalization of the agro-industrial cycle has stimulated the search for a possible exploitation of fruit residue, from a circular bioeconomy perspective. Pomegranate peels have been widely used for the treatment of different pathologies such as inflammation, ulcers, infections, and brain ischemia, while the seeds can be used to produce a high-quality oil [5,6]. Recently, our group reported an herbal mixture made from propolis, pomegranate peels, and grape pomace as an active anti-inflammatory agent in an in vivo rheumatoid arthritis, model highlighting its possible use as a new natural product-based formulation against this disease [7].

In several areas of South Italy, there is a long-held tradition concerning the cultivation, in parks and public or private gardens, of pomegranates used as ornamental plant, and for juice and eating. Recently, due to the discovery of the healthy and nutritional promoting properties of the fruit, its cultivation has been increased, yielding a high quantity of bio-waste. Although the pomegranate cultivation in the internal areas of South Italy could produce notable economic and commercial income with interesting development opportunities, no studies have been reported until now on the pomegranate cultivars collected from these Italian areas. Therefore, to valorize them as by-products, pomegranate peels from eight cultivars of South Italy were selected and subjected to a quali-quantitative comparisons for their secondary metabolite contents. Among the investigated cultivars, ‘Granato di Aiello del Sabato’, a local accession of Campanian internal areas with a good yield from an agronomic point of view as it adapts perfectly to this area environment, was selected with the aim to promote its cultivation by local farmers also through the valorization of its main by-products. Furthermore, considering the high interest in pomegranate by-product biological activity, extracts from the peels were evaluated for their radical scavenger activity by DPPH and ABTS^+^-based and in-cell antioxidant activity assays. Subsequently, the in vitro anti-inflammatory potential was also investigated by monitoring their effect on the secretion of three typical markers of inflammation (IL-1β, IL-6, and TNF-α) in human macrophages. Finally, several bioinformatic analyses were carried out to achieve a deeper understanding of the chemical profiles characterizing the different cultivars as well as to identify potential biochemical markers associated with the bioactivities. Specifically, to the best of our knowledge, this is the first study on the chemical and bioactive profile of the ‘Granato di Aiello del Sabato’ cultivar.

## 2. Materials and Methods

### 2.1. Samples

The fourteen pomegranate fruit peels, M1-M14, were obtained in autumn 2020 from eight cultivars and different collection sites as reported in Table 1. The whole fruits were separated into arils and peels, and the peels were stored at −22 °C until the extraction procedure.

### 2.2. Reagents

Ultra-pure acetonitrile, water, methanol, and formic acid for LC-MS analysis were purchased from Romil Ltd. Pure Chemistry (Cambridge, UK). Solvents for extraction were purchased from Sigma Chemicals Company (Milan, Italy). For quali-quantitative analysis, the following standards were used: punicalin and punicalagin from PhytoLab GmbH & Co. KG (Vestenbergsgreuth, Germany), ellagic acid, gallic acid, apigenin 7-*O*-glucoside, and cyanidin 3-*O* glucoside were obtained from Sigma Chemicals Company (Milan, Italy). THP-1 (human leukemia monocytic) and HaCat (human epidermal keratinocytes) cell lines were purchased from American Type Cell Culture (ATCC) (Rockville, MD, USA). DMEM (Dulbecco’s Modified Eagle Medium) and RPMI 1640 (Roswell Park Memorial Institute Medium), Fetal Bovine Serum (FBS) were purchased from GIBCO (Life Technologies, Grand Island, NY, USA). Phorbol-12-myristate-13-acetate (PMA), MTT [3-(4,5-dimethylthiazol-2-yl)-2,5-diphenyl tetrazolium bromide] were purchased from Sigma Aldrich (St. Louis, MO, USA). OxiSelect™ Cellular Antioxidant Activity Assay Kit was purchased from CellBiolLabs (San Diego, United States). Human IL-1β, IL-6, TNF-α, and ELISA Kits were purchased from Diaclone (Besançon Cedex, France).

### 2.3. Peels Extraction

5 g of each *P. granatum* dried peel cultivar were extracted with EtOH-H_2_O 7:3. The extraction was performed using a 320 W Ultrasonic bath (Branson 2510E-MTH, Bransonic^®^, Milan, Italy) for 15 min. The amount of solvent used was 1:10 (*w*/*v*). The extracts, after filtration, were dried under vacuum, frozen, and lyophilized to remove the exceeded water and stored at 4 °C for further analysis.

### 2.4. Qualitative Profiling of Pomegranate Peels Hydroalcoholic Extracts

All fourteen hydroalcoholic extracts were dissolved in H_2_O-MeOH at a ratio of 4:1 to obtain a final concentration of 5 mg/mL and subjected to HR-ESI-LC-MS/MS analysis. A Luna^®^ C_18_ 150 × 2 mm, 3 µm (100 Å) column (Phenomenex^®^, Castel Maggiore, Bologna, Italy) was employed using H_2_O acidified by 0.1% formic acid *v*/*v* (solvent A) and CH_3_CN (solvent B) with the following linear gradient as the elution method: solvent B from 5 to 50% over 50 min to 50 to 100% in 10 min. The flow rate was set to 0.2 mL/min and the column oven was set to 40 °C. Q Exactive™ Hybrid Quadrupole-Orbitrap™ Mass Spectrometer (Thermo Fischer Scientific Inc., Darmstadt, Germany) was operated in the negative ion mode coupled with the Thermo Scientific UltiMate 3000 UHPLC system. The identification of specialized metabolites was based on accurate MS values and an MS/MS spectra, aided by the injection of standard compounds and comparison with data from previous literature [8].

### 2.5. Quantitative Analysis

The quantitative determination of gallic acid, cyanidin 3-*O*-glucoside, delphynidin 3-*O*-glucoside, ellagic acid, punicalin, and punicalagin was carried out by ABSCIEX API 6500 QTRAP^®^ Mass Spectrometer coupled with a Nexera X2 UPLC Shimadzu system in both the positive and negative ion modes. A Luna^®^ Omega 100 × 1.6 mm, 3 µm (100 Å) column (Phenomenex^®^, Castel Maggiore, Bologna, Italy) was employed using H_2_O acidified by 0.1% formic acid *v/v* (solvent A) and CH_3_CN (solvent B). Two different gradient methods were set up for anthocyanins and for the other compounds. The first method provides a linear gradient from over 12 min followed by a faster gradient until reaching 100% of B in 3 min. The second gradient started from 5% of B and reached 30% in 18 min, this was followed by a faster gradient until 100% of B was reached. In both methods, the flow rate was set to 0.25 mL/min and the column oven was set to 30 °C. Analyses were performed in positive and negative ion modes and, for each analyte, the mass parameter was optimized using a standard molecule. The calibration curves for each compound were obtained at a concentration range from 10 ng/mL to 5 μg/mL. The linearity of the instrumental response in the analyzed concentration range was confirmed for each compound, as inferred by the following fitting curve parameters: gallic acid, y = 1.5 × 10^3^ x + 1.9 × 10^5^, R^2^ = 0.9922; cyanidin 3-*O*-glucoside,y = 3.8 × 10^4^ x – 3.0 × 10^6^, R^2^ = 0.9966; delphynidin 3-*O*-glucoside y = 5.0 × 10^3^ x + 9.7 × 10^4^, R^2^ 0.9986; ellagic acid y = 1.5 × 10^4^ x + 1.0 × 10^6^, R^2^ 0.9984; punicalin, y = 1.3 × 10^3^ x −3.6 × 10^4^, R^2^ = 0.9992, and punicalagin y = 3.9 × 10^3^ x − 7.6 × 10^5^, R^2^ = 0.9968. For all of the compounds the lowest concentration of each calibration curve was clearly higher than respective LOQ, as the signal-to-noise ratios observed at those points were between 8 to 12.

### 2.6. DPPH Assay

The DPPH (2,2′-diphenyl-1-picrylhydrazyl radical) assay is a spectrophotometric technique where the radical cation reacts with hydrogen donors [9]. The violet color that shows the DPPH in the solution at 515 nm is decolorized by the presence of antioxidants. A stock solution of 10 mM DPPH was freshly prepared in methanol and diluted until an absorbance of 1 OD at 515 nm was reached. Samples were assayed in the presence of a 0.15 mM final concentration of DPPH in 100% methanol. Extracts were diluted between 500 and 2000-fold; the reaction was allowed to proceed for a maximum of 30 min in the dark at room temperature, and then the decrease in absorbance at 515 nm was measured. Different Trolox µM concentrations (0–100 µM-*X*-axis) were incubated in the presence of the DPPH radical, and its absorbance was measured at 515 nm (*Y*-axis). A calibration curve was constructed with Trolox concentrations and Abs at 515 nm. All solutions were used on the day of preparation, and all determinations were carried out in triplicate. Millimolar concentrations of Trolox equivalents (TE) of dry extract were quantified using the linear regression equation as follows: extract TE µM = [(Abs 515 nm–1.1057)/(−0.006)]. Then, the appropriate dilution factor was applied to calculate the millimolar TE of the extract at 10 mg/mL.

### 2.7. ABTS Assay

The ABTS radical cation decolorization assay is based on the reduction of ABTS^+•^ radicals by the antioxidants included in an extract [10]. For the study, the ABTS^+•^ solution was diluted in PBS (Phosphate Buffer Saline) to an absorbance of 0.7 (±0.02) at 734 nm. After the addition of 100 μL of extract solutions to 100 μL of ABTS^+•^ solution, the absorbance reading was taken at 30 °C for 10 min after initial mixing. All solutions were used on the day of preparation, and all determinations were carried out in triplicate. Samples were compared to known concentrations of Trolox standards, a water-soluble analog of tocopherol (Vitamin E), which is a very strong antioxidant and commonly used to measure antioxidant capacity. Different Trolox µM concentrations (0–50 µM-*X*-axis) were incubated in the presence of the ABTS radical, and its absorbance was measured at 734 nm (*Y*-axis). Micromolar concentrations of Trolox equivalents (TE) of dry extract were quantified using the linear regression equation as follows: extract TE µM = [(Abs 734 nm–0.8594)/(−0.0156)]. Then, the appropriate dilution factor was applied to calculate the millimolar TE of the extract at 10 mg/mL.

### 2.8. Cell Viability Assay

A cell viability assay was performed on THP-1 (human acute monocytic leukaemia cell line) and HaCat (human epidermal keratinocytes). THP-1 were plated in 96-well plates at a cell density of 1 × 10^5^ cells/well and differentiated in the THP-1 macrophage attached cell line by treating with 100 nM of phorbol-12-myristate-13-acetate (PMA) for 24 h. THP-1 cell differentiation was enhanced by removing the PMA-containing media and adding fresh media for 24 h. Then, they were incubated for 24 h in the presence of the extracts at concentrations in the range 25–100 μg/mL. HaCat were plated in 96-well plates at a cell density of 1 × 10^4^ cells/well. Then, the cells were incubated for 48 h in the presence of extracts at concentrations of 100 μg/mL. For both cell lines, the number of viable cells was quantified by the MTT [3-(4,5-dimethylthiazol-2-yl)-2,5-diphenyl tetrazolium bromide] assay. Absorption at 550 nm for each well was assessed using Multiskan GO (Thermo Scientific). Experiments were performed in technical triplicates.

### 2.9. Antioxidant Activity in M0 Macrophages

Using the OxiSelect™ Cellular Antioxidant Activity Assay Kit, the cells were differentiated, as reported above. Then, they were treated with a DCFH-DA probe solution in association with a quercetin standard (0.125–2 mM) or an extract sample (50 µg/mL) and incubated for 1 h. After washing with DPBS 1X buffer, the free radical initiator solution was incubated and immediately the plates were read on a fluorescence microplate reader using an excitation wavelength of 480 nm and an emission wavelength of 530 nm. The reading was performed in the time interval 0–60 min with a reading every 5 min. The analysis was firstly performed evaluating the AUC value (area under the curve) for each sample and then the CAA value (cellular antioxidant activity) was calculated as follows: [CAA Units = 100 − (AUCAntioxidant/AUCControl) × 100] [11].

### 2.10. Cytokine Production and Enzyme-Linked Immunosorbent Assay (ELISA)

The THP-1 were differentiated in the THP-1 macrophage attached cell line by treating with 100 nM of PMA for 24 h. THP-1 cell differentiation was enhanced by removing the PMA-containing media and adding fresh media for 24 h. The cells were incubated with extracts (100 µg/mL) with and without LPS (0.1 µg/mL) for 24 h. The conditioned medium was collected and analyzed by an Enzyme-Linked Immunosorbent Assay (ELISA). The assays were performed according to manufacturer instructions to quantify the release of inflammatory cytokines (IL-1β, IL-6, and TNF-α). The values were normalized to the LPS sample and reported as percentages. Experiments were performed in technical triplicates.

### 2.11. Bioinformatics Analyses

Statistical analyses of chemical and bioactivity data have been carried out as previously reported [12], using an ANOVA coupled to a pairwise Tukey’s *t*-test performed by the PAST software. Heatmaps and hierarchical clustering (HCL) were performed using the Morpheus as reported before, whereas correlation analyses were done as previously described [13], but only considering negative significant (*p* ≤ 0.05) correlations between bioactivities and chemical compounds.

## 3. Results

### 3.1. LC-MS/MS-Based Quali-Quantitative Analysis

**Figure 1:**
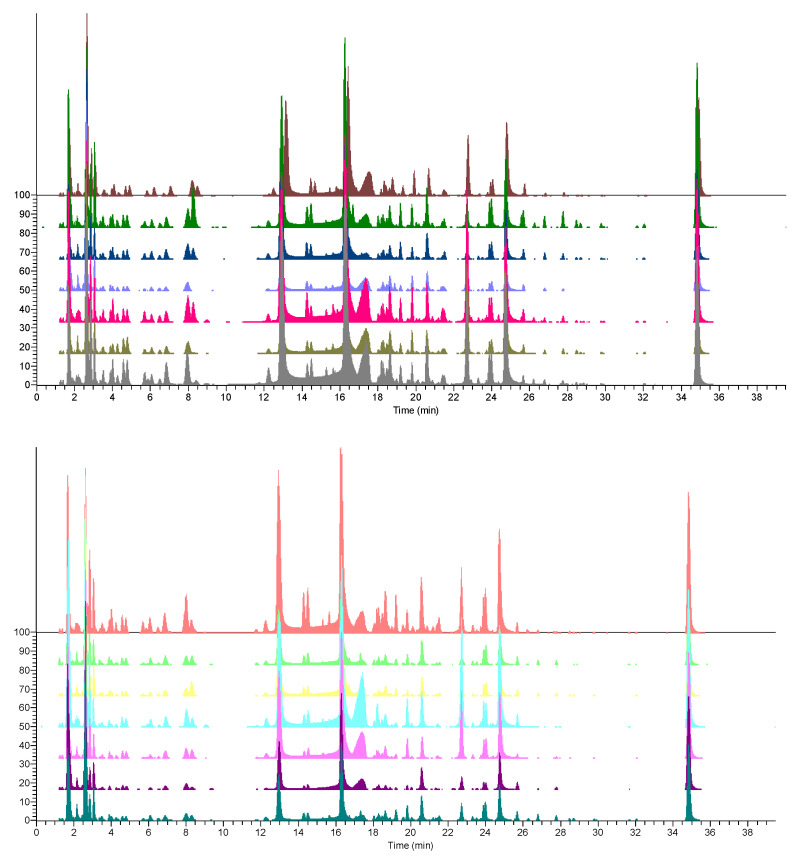
UHPLC-HR-ESI-MS profiles of the fourteen peel samples M1–M14 (M1 the first up, M-14 the last down) in the negative ion mode.

Eight cultivars of *P. granatum* from different collection areas (Table 1) were obtained from Campanian plant breeders and companies, including those widely cultivated to produce pomegranate juice such as ‘Dente di Cavallo‘ and ‘Wonderful‘. All pomegranate accessions come from the same 2020 collection year, and the fruits were harvested at complete maturation. Their peels were subjected to hydroalcoholic ultrasound-assisted extraction. LC-MS/MS analysis (Figure 1 and Figure S1) of the resulting extracts revealed that all fourteen samples displayed almost superimposable profiles with only small differences.

In particular, according to MS data, retention time, and comparison with available pure standards, 45 ellagitannins, consisting of many polyhydroxyphenoyl groups (such as hexahydroxyphenoyl-HHDP) also characterized by the presence of a C–C linkage between galloyl units and 10 flavonoids, belonging to flavonol, flavone, and catechin classes, were tentatively identified (Table 2, Figures S2 and S3). Moreover, among anthocyanins, cyanidin 3-*O-*glucoside was detected in all the samples, whereas delphinidin 3-*O*-glucoside was found only in M13 (Table 3, Figure S4). These results were in agreement with previous reports of the specialized metabolite composition in pomegranate peels [14,15,16,17,18,19].

The most representative compounds underwent quantification through a LC-MS/MS multiple reaction monitoring (MRM)-based method. Although the qualitative profile of the different accessions was almost superimposable, there was variability in their phenolic content. The results obtained (Table 3) showed that punicalagin was the most abundant compound in all samples, with sample M7 being the richest. Ellagic acid and punicalin were the second and the third most represented compounds in all extracts, respectively, while gallic acid was present in lower amounts. The M13 extract showed a quantitative profile quite different from the others; in fact, it was richer in anthocyanins, according to the different peel color, but showed a lower quantity of the tannin compounds. This evidence suggested a slightly different secondary metabolism for the ‘Parfianka’ species compared to the others. To the best of our knowledge, this is the first time the peel composition of the M5 cultivar, ‘Granato Aiello del Sabato’, was investigated, being one of the highest phenolic compounds among the analyzed species.

### 3.2. Antioxidant Activity

The pomegranate peel extracts were analyzed for their antioxidant activity using DPPH and ABTS^+^-based assays. Using this approach, the antioxidant capacity of each extract was estimated in terms of Trolox equivalent antioxidant capacity (TEAC) and reported as the average of those resulting from at least three independent experiments (Table 4, Figure S5A) [21,22,23,24]. Different antioxidant activities were observed among pomegranate cultivars. Significant differences in the results of the DPPH assay were registered, with the activity values ranging from 40.1, obtained for M13 ‘Parfianka’, to 72.8 and 70.0, measured for M3 ‘Wonderful’ Sicignano degli Alburni and M14 ‘Wonderful’ Grottaminarda, respectively. Interestingly, also ABTS activity data showed that M14 ‘Wonderful’, Grottaminarda accession and M3 ‘Wonderful’ Sicignano degli Alburni accession displayed the highest value.

**Figure 2:**
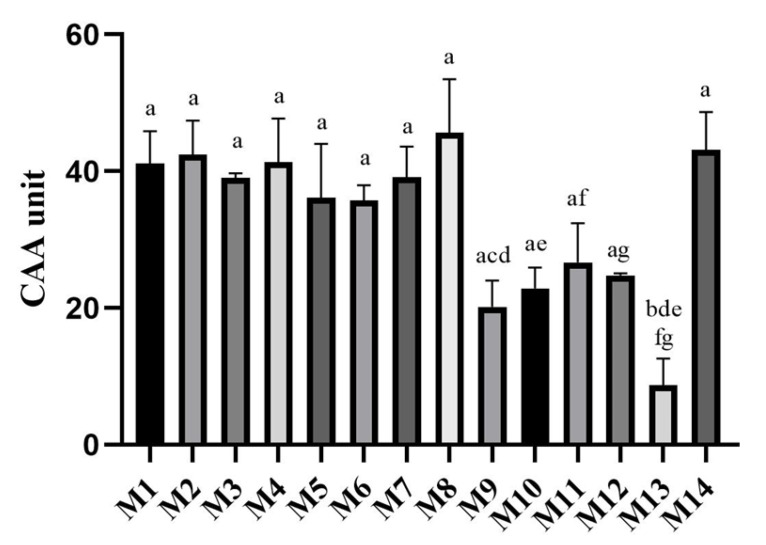
Cellular antioxidant activity as CAA values of all pomegranate peels (*n* = 2). Different letters within each column indicate statistically significant differences at *p* < 0.05 in an ANOVA + Tukey’s pairwise *t*-test analysis.

A further evaluation of the antioxidant activity was carried out through an in-cell assay performed on the human acute monocytic leukemia cell line THP-1-derived M0 macrophages. Preliminarily, the cytotoxic activity of the pomegranate peel extracts towards these cells was evaluated by an MTT assay. The cells were incubated with the extracts for 48 h at a concentration of 100 μg/mL and no cytotoxicity was observed. Once the cytotoxic effect was verified, the antioxidant activity of pomegranate peel extract was evaluated using OxiSelect™ Cellular Antioxidant Activity Assay Kit (Figure 2). Compared to quercetin, used as a positive control, most of the tested extracts also showed an interesting antioxidant effect in cells. However, the extracts M9, M10, M11, M12, and, most importantly, M13, were clearly and significantly less effective than the others.

### 3.3. Anti-Inflammatory Activity

**Figure 3:**
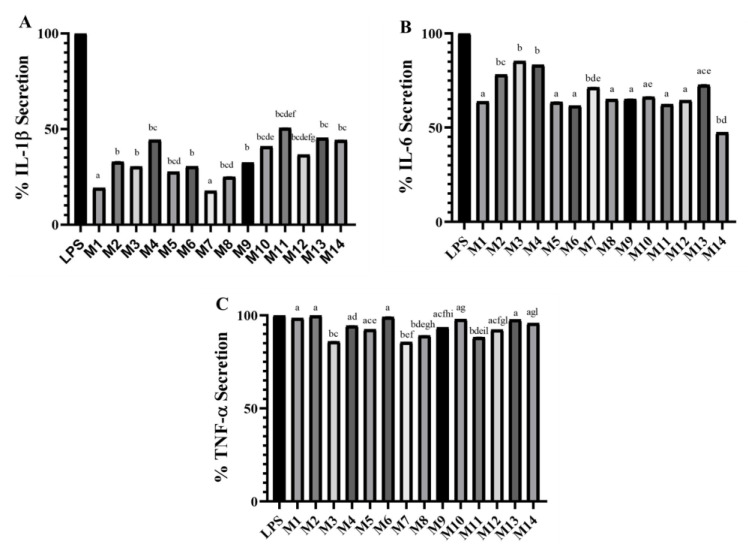
Percentage of secretion of IL-1β (**A**), IL-6 (**B**), and TNF-α (**C**) from LPS-stimulated THP-1-derived macrophage cells after 24 h of treatment with M1-M14 extracts. Different letters within each column indicate statistically significant differences at *p* ≤ 0.05 in an ANOVA + Tukey’s pairwise *t*-test analysis.

Subsequently, the anti-inflammatory activity of pomegranate peel extract was evaluated. Since pro- and anti-inflammatory cytokines play a key role in the pathogenesis and evolution of the inflammatory state of rheumatoid arthritis, three typical markers of rheumatoid arthritis were monitored: IL-1β, IL-6, and TNF-α (Figure 3 and Figure S5B). Although their appearance is temporally different, especially in the early course of pathology, they are considered key driving molecules [25]. The extracts were tested on THP-1-derived macrophage cells co-stimulated with LPS. All pomegranate peel extracts revealed potential anti-inflammatory activity, as demonstrated by the observation that all the treatments significantly reduced the secretion of IL-1β and, to a lesser extent, that of IL-6. Instead, TNF-α was only slightly modulated by the peel extract treatment (Figure 3). In particular, M1 and M7 samples were able to inhibit the IL-1β secretion by about 80%, while M5 and M8 inhibited it by about 75%, compared to LPS (Figure 3A). The lowest activity was displayed by M4 and M11 samples (about 55% of reduction). Regarding the IL-6 secretion inhibition, the M14 sample was the most active (55% reduction), while M1, M5, M6, M8, M9, and M11 showed a reduction of about 40% (Figure 3B). Based on these results, IL-6 and IL-1β seem to be the pro-inflammatory cytokines most specifically modulated. In particular, IL-1β appears as the most suitable biomarker to evaluate the potential anti-inflammatory activity of pomegranate peel extract, thus confirming what has already been shown regarding the anti-inflammatory activity of this fruit on human chondrocytes [26,27].

### 3.4. Bioinformatics Analyses

**Figure 4:**
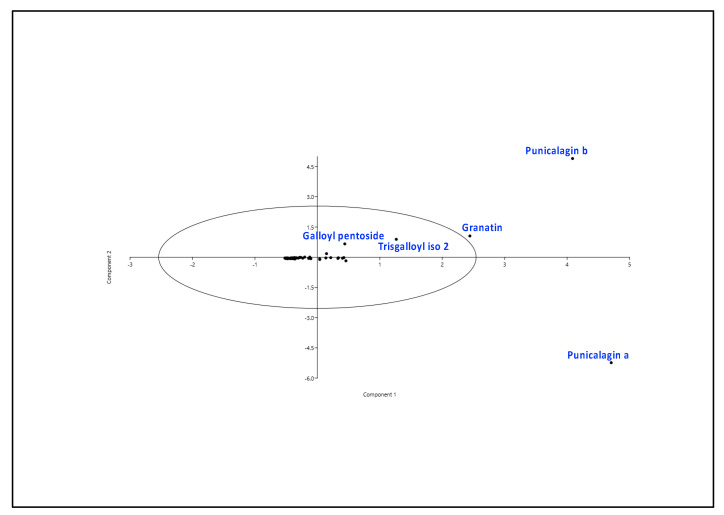
Principal component analysis (PCA) of the metabolites of M1-M14 peel samples. Component 1 and 2 explained, respectively, 77.8% and 14.4% of the total variance.

In order to achieve a more detailed understanding of the chemical and bioactivity data under study, a series of bioinformatics approaches were carried out. First of all, multivariate analyses (principal component analyses, PCAs) were performed, either at variety or metabolite levels (Figure 4 and Figure S4), while the latter was not able to clearly discriminate the different varieties (which clustered all together, Figure S6). In agreement with what reported in Section 3.1, the former (variety) highlighted a group of compounds responsible for the total variance of the dataset. More specifically, punicalagin a and b, granatin, and to a lesser extent, trisgalloyl iso 2 and galloyl-pentoside resulted in the most variable metabolites among the pomegranate genotypes (Figure 4). Notably, components 1 and 2 explained more than 90% of the total variance. We also measured PCA loadings and scores (Tables S1 and S2A,B, Figures S7 and S8), which evidenced, except for M4, positive contributions to components 1 and 2 (PC1, PC2) and a positive and negative influence of M10 on, respectively, PC1 and PC2 (Table S1A,B, Figure S7). PCA and loading scores also confirmed the role of two of the aforementioned metabolites (punicalagin a and b) as the metabolites driving the variance of the whole chemical dataset under study.

**Figure 5:**
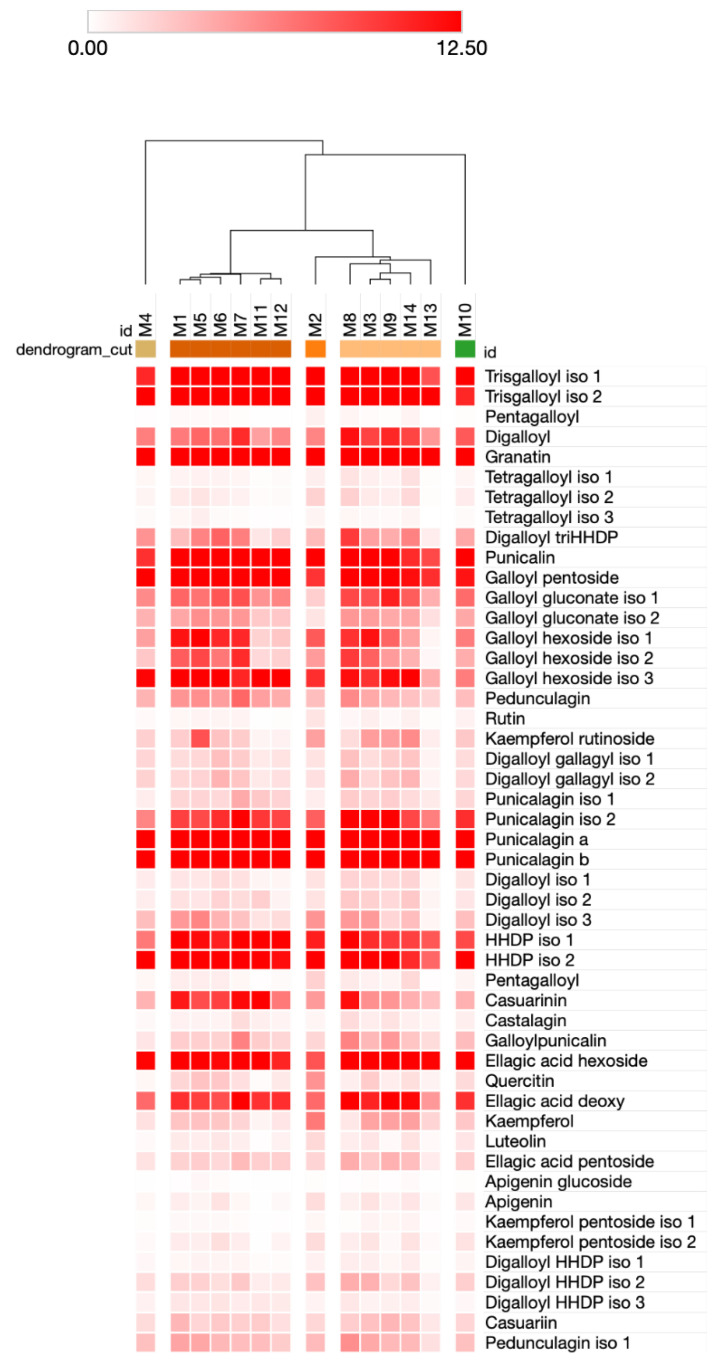
Hierarchical clustering (HCL) analysis of chemical data from peel pomegranate samples. HCL was calculated on columns by applying the One Minus Pearson correlation with the average linkage algorithm and evidencing the presence of specific clusters. Increasing intensity of the red color is directly proportional to compound intensity. The data represent, for each, LC-MS signal intensities and are expressed as average values/100,000,000.

Subsequently, a hierarchical clustering (HCL) analysis was exploited as an alternative strategy to evaluate the differences among the pomegranate cultivars under study at global chemical profiling levels (Figure 5). Notably, HCL was able to clearly separate the different genotypes by highlighting the presence of five clusters. More specifically, a distinct attitude of M14 and to a lesser extent, M10, as the varieties with the more marked profiling was found, which placed farthest compared to the others at the left and right sides of the HCL, respectively. On the contrary, all remaining cultivars were distributed into two well recognizable groups, one composed of M1, M5, M6, M7, M11, and M12, and the other including M2, M8, M3, M9, M14, and M13 (Figure 5). It was interesting to notice that genotypes belonging to the same variety mostly grouped together: for instance, the four ‘Dente di Cavallo’ samples (M1, M6, M7, and M11) were all found in the first group, whereas the two additional cultivars were placed in the second group together with the two ‘Wonderful’ cultivars (M3, M14). Thus, HCL resulted a powerful and effective method to infer the chemical differences among the pomegranate cultivars under study, and proved the existence, at least for ‘Dente di Cavallo’, of either genotype- (M1, M6, M7, M11) and genotype x environment-based (M3, M14) effects (Figure 5).

A detected bioactivity can be associated with the presence of a few or a group of molecules in a specific peel extract [28]. In order to elucidate whether specific compounds could be responsible for the antioxidant and anti-inflammatory activity of pomegranate peel extracts and which compounds they were, we performed a correlation analysis, calculating the Pearson correlation coefficients (ρs) between each metabolite and the analyzed bioactivities (Table 5 and Table S3A,B). More specifically, to highlight relationships of interest, only negative significant correlations were considered (corresponding to opposite tendency between metabolite and bioactivity data). Overall, it was not possible to find a large number of significant correlations, thus suggesting the absence, at least on a mathematical basis, of a phytocomplex-derived effect. However, this analysis allowed identifying compounds displaying significant ρs towards the antioxidant and anti-inflammatory activities, notable examples include granatin, trisgalloyl iso 2, and ellagic acid, displaying significant negative correlations towards all antioxidant activities (Table 5A and Table S3A). Similarly, although at a higher extent, a group of compounds showed significant negative correlations towards anti-inflammatory activities, including galloyl hexoside iso 2, pedunculagin, and punicalagin iso 2, being negatively correlated with IL-1β and TNF-α; and punicalagin iso 2, ellagic acid deoxy, and ellagic acid pentoside, displaying significant negative correlations towards IL-1β IL-6, and TNF-α (Table 5B and Table S3B).

## 4. Discussion

The recovery of agricultural and industrial waste is now recognized as an essential process due to its great economic importance and the reduction of the environmental impact of production processes. The opportunity to transform materials whose disposal involves costs and risks for the environment and population into a source of raw materials could be crucial in those regions, such as the internal areas of South Italy, which are still underdeveloped economically. From this point of view, the possible recovery of pomegranate production chain waste seems to have all the optimal characteristics to be carried out in a productive and advantageous way. Indeed, the main pomegranate by-product is the pericarp of the fruit, and several studies have suggested these peels as a source of bioactive phytocomplexes [29] and proposed their use for several applications [30,31]. However, in order to achieve real feasibility of a waste recovery process aimed at obtaining extracts rich in bioactive molecules, it is advisable that the recovery cost be compatible with the extraction process yield and the quality of the obtained products. Since multiple factors (for example, genetic, geographic, and climatic) have critical effects on the production of secondary metabolites [32,33], it is important to evaluate their effect on the characteristics and quality of by-products. Many studies support the antioxidant and anti-inflammatory effects of the phytochemicals from different *P. granatum* cultivated varieties [34,35]. In this report, the results of a study aimed at evaluating the possibility of using pomegranate peels from South Italy’s internal areas as a source of extracts with antioxidant and anti-inflammatory activities were pointed out. The rich phenolic compound composition obtained didn’t show a great deal of variability among different pomegranate accessions (Table 1), suggesting that environmental conditions, such as climate or soil composition (clayey or sandy), slightly affect their secondary metabolism. In fact, the comparison of the phenolic compound composition within the different cultivars and accessions of pomegranate and the literature data could be a tricky process due to various factors such as cultivar type, weather conditions, soil composition, and ripeness [33]. Our analyses have confirmed that pomegranate peels contain secondary metabolites of great interest for health and chemoprevention, nutritional, or cosmetic use, such as ellagitannins, flavonoids, and anthocyanins [36]. As expected, the presence of these molecules provided the extracts with a significant antioxidant capacity, appreciable both through in vitro assays and studies on human cells.

The inflammation provokes a condition of oxidative stress that reduces the antioxidant capacity of cells, causing cellular damage. This process is mediated by a variety of factors that contribute to both the release of cytokines and the expression of pro-inflammatory mediators, contributing to the exacerbation of the pro-inflammatory process [7]. Thus, the anti-inflammatory activity of the peel extracts was investigated in cells. Firstly, all the extracts were assayed by the MTT test on THP-1 and HaCat cell lines to verify they did not affect cell viability. Among the factors involved in the inflammation process cytokines play a pivotal role; thus, the secretion of three typical markers, IL-1β, IL-6, and TNF-α, in cells treated with pomegranate peel extracts were investigated. The high modulation of IL-1β, exerted by the majority of the extracts is of high interest due to the critical role of this cytokine in the pathogenic mechanism. However, the modulation of IL-6 is also critical, especially in the systemic manifestations in the acute phase of the inflammatory process [27]. Therefore, the observed ability of pomegranate peel extract to inhibit IL-1β and IL-6 secretion supports their possible use to address inflammation at different stages. On the other hand, the obtained results corroborate the idea that these cytokines could be considered as putative targets for an early intervention on inflammatory pathologies, such as rheumatoid arthritis [7]. A comparison between the peels did not allow us to highlight substantial differences with regards the composition of secondary metabolites of the respective extracts and, consequently, no statistically significant differences in biological activity were observed between the various samples. In fact, different approaches to statistical and multivariate analysis of the various experimental data collected, failed to identify a clear tendency between pomegranate varieties and bioactivities. However, using correlation analyses of chemical composition and bioactivity data, it was possible to highlight, for some metabolites, a potential positive contribution to the detected activities, either at antioxidant or anti-inflammatory levels. This evidence confirms once again the importance of using suitable bioinformatics tools to study complex systems such as phytocomplexes, whose characteristics cannot be considered as the mere average of the properties of the individual components [37].

## 5. Conclusions

Eight cultivars of pomegranate peel, collected from different South Italian accessions in the same harvesting year, were chemically compared. The composition of their bioactive specialized metabolites was investigated, with a focus on ellagitannins and flavonoids, including anthocyanins. The main components were also quantified. Qualitative profiles obtained for both ellagitannins and flavonoids were almost superimposable; on the other hand, the quantitative analysis showed some variability. Antioxidant data were generally good for the majority of the accessions. Results of anti-inflammatory activity confirmed that pomegranate by-product extracts could be particularly interesting for the prevention of inflammation since they strongly inhibited IL-1β, while reducing IL-6 secretion. Overall, these data suggested a potential synergic antioxidant and anti-inflammatory effect of the pomegranate peel extracts. PCA analysis highlighted punicalagin a and b, granatin, trisgalloyl iso 2, and galloyl pentoside as the metabolites mainly responsible for the total variance, whereas HCL evidenced the presence of two distinct groups, with M4 and M10 varieties showing a divergent attitude. Finally, a series of compounds, including galloyl- and ellagic acid-based were found to be highly correlated with antioxidant and anti-inflammatory activities. These results open the possibility for the introduction of pomegranate peels into the South Italian by-circular economy in order to reduce their quantity and environmental impact while increasing the cultivation in these unexploited areas. Particularly, the M5 accession, ‘Granato di Aiello del Sabato’, investigated here for the first time, having one of the highest phenolic content and one of the highest ability in reducing IL-1β and IL-6 secretion from macrophages, could be the autochthonous internal areas of Campania region cultivar most suitable for an extensive exploitation. This investigation could ensure the transmission to the farmers and the sustainability and conservation of this genetic material, making it a peculiarity for local companies and obtaining its germplasm protection and constant propagation.

## Figures and Tables

**Table 1:** Cultivars and collection area of the fourteen *Punica granatum* samples.

Sample	Collection Area	Cultivars	Company
M1	Benevento, Campania region	Dente di cavallo	Caruso
M2	Palomonte, Campania region	Dente di cavallo	Rosso Granato
M3	Sicignano degli Alburni, Campania region	Wonderful	Rosso Granato
M4	Palomonte, Campania region	Wonderful One	Rosso Granato
M5	Aiello del Sabato, Campania region	Granato di Aiello del Sabato	Giovomel
M6	Pietrelcina, Campania region	Dente di cavallo	Salgliocca
M7	Acri, Calabria region	Dente di cavallo	Cofone
M8	Tursi, Basilicata region	Dente di cavallo	Francolino
M9	Forchia, Campania region	Hicaz	ErarslanFarm
M10	Caserta, Campania region	Wonderful precoce	Luce
M11	Altavilla silentina, Campania region	Dente di cavallo	De Matteis
M12	Altavilla silentina, Campania region	Mollar de Elche	De Matteis
M13	Telese, Campania region	Parfianka	Troiano
M14	Grottaminarda, Campania region	Wonderful	Bruno

**Table 2:** UHPLC-HR-ESI-MS/MS data of compounds detected in pomegranate peels M1-M14.

	*t*_R_ (min)	[M-H]^2−^/[M-H]^−^	MS^2^	Compound	MSI Status ^a^
1	2.1	481.0632	300.9991, 275.0185	HHDP ^b^-hexoside 1	2
2	2.8	481.0632	300.9991, 275.0185	HHDP-hexoside 2	2
3	3.0	331.0680	271.0449, 211.0239, 169.0143	Galloyl-hexoside 1	2
4	3.5	331.0680	271.0449, 211.0239, 169.0143	Galloyl-hexoside 2	2
5	3.9	649.0705	300.9991, 497.0575	Galloyl-HHDP-gluconate	2
6	4.0	633.0752	481.0630, 300.9991, 249.0407, 275.0185	HHDP-galloyl-hexoside	2
7	4.2	169.0138	125.0249	Gallic acid	1
8	4.3	331.0680	271.0449, 211.0239, 169.0143	Galloyl-hexoside 3	2
9	4.4	483.0798	331.0671, 313.0566, 169.0143	Digalloyl-hexoside	2
10	4.6–4.8	781.0555	721.0330, 600.9907, 475.0361	Punicalin	1
11	4.9	483.0798	331.0671, 313.0566, 169.0143	Digalloyl-hexoside isomer	2
12	5.7	633.0752	481.0630, 300.9991, 249.0407, 275.0185	HHDP-galloyl-hexoside isomer	2
13	6.0	391.0321/783.0712	765.0588, 721.0330, 481.0630, 300.9991	Casuariin	2
14	6.1	541.0272/1083.0630	807.0330, 721.0330, 600.9907, 510.0260, 275.0185	Punicalagin isomer 1	2
15	6.5	649.0705	300.9991, 497.0575	Galloyl-HHDP-gluconate	2
16	6.8	541.0272/1083.0630	807.0330, 721.0330, 600.9907, 510.0260, 275.0185	Punicalagin isomer 2	2
17	7.1	483.0798	331.0671, 313.0566, 169.0143	Digalloyl-hexoside isomer	2
18	7.2	466.0299/933.0679	915.0552, 781.0550, 721.0330, 600.9907, 300.9991	Castalagin	2
19	7.8	391.0321/783.0712	631.0589, 481.0630, 300.9991	Pedunculagin	2
20	7.9	707.0647/1415.1368	783.0690, 613.0480, 633.0739, 300.9991	Di (HHDPgalloyl-hexoside)-pentoside	2
21	8.3	305.0675	125.0249, 137.0250	Gallocatechin	2
22	8.4	466.0299/933.0679	781.0550, 631.0589, 450.9945, 425.0153, 300.9991, 275.0185	2-*O*-Galloylpunicalin	2
23	12.9	541.0272/1083.0630	781.0550, 600.9907, 300.9991, 275.0185	Punicalagin A	1
24	14.3	391.0318/783.0708	631.0589, 481.0630, 300.9991	Pedunculagin isomer	2
25	14.5	951.0767	907.0867, 783.0690, 605.0800, 481.0630, 300.9991	Trisgalloyl HHDP-hexoside	2
26	15.3	632.0670/1265.1423	783.0690, 481.0630	Pedunculagin-I-der	2
27	16.2	392.0392/785.0859	300.9991, 275.0185, 249.0407, 169.0143	Digallolyl HHDP-hexoside	2
28	16.3	541.0272/1083.0630	781.0550, 600.9907, 300.9991, 275.0185	Punicalagin B	1
29	17.4	799.0657	781.0550, 479.0476, 300.9991	Granatin A	2
30	18.2	467.0374/935.0822	917.0711, 783.0690, 633.0739, 571.0740	Galloyl-bis-HHDP-hexoside (casuarinin)	2
31	18.3	400.0371/801.0814	649.0681, 499.0724, 347.0623, 300.9991	Punigluconin	2
32	18.4	783.0718/1567.1511	765.0588, 300.9991	Digalloyl triHHDP-dihexoside	2
33	19.2	392.0392/785.0859	300.9991, 275.0185, 249.0407, 169.0143	Digallolyl-HHDP-hexoside	2
34	19.2	542.0355/1085.0795	783.0690, 765.0588, 300.9991	Digalloyl-gallagyl-hexoside	2
35	19.6	542.0355/1085.0795	783.0690 765.0588, 300.9991	Digalloyl-gallagyl-hexoside isomer	2
36	19.8	633.0752	481.0630, 300.9991, 249.0407, 275.0185	HHDP galloyl-hexoside isomer	2
37	20.6	463.0530	300.9991	Ellagic acid hexoside	2
38	22.7	951.0767	933.0668, 765.0588, 613.0480, 445.0425, 300.9991	Trisgalloyl HHDP-hexoside	2
39	23.6	787.1035	635.0883, 617.0779, 465.0671, 447.0562	Tetragalloyl-hexoside isomer 1	2
40	23.7	392.0392/785.0859	300.9991, 275.0185, 249.0407, 169.0143	Digallolyl HHDP hexoside	2
41	23.8	433.0428	299.9917, 300.9991	Ellagic acid-pentoside	2
42	24.0	447.0580	300.9991, 300.9991	Ellagic acid-deoxyhexoside	2
43	24.2	787.1035	635.0883, 617.0779, 465.0671, 447.0562	Tetragalloyl-hexoside isomer 2	2
44	24.6	787.1035	635.0883, 617.0779, 465.0671, 447.0562	Tetragalloyl-hexoside isomer 3	2
45	24.7	609.1458	301.0356	Rutin	1
46	24.8	300.9995	257.0094, 229.0146, 185.0249	Ellagic acid	1
47	25.5	463.0902	301.0356	Quercetin-hexoside	2
48	26.3	939.1158	787.1030, 635.0883	Pentagalloyl-hexoside	2
49	26.8	593.1534	447.0948, 285.0400	Kaempferol-rutinoside	2
50	27.8	447.0948	285.0400	Kaempferol-hexoside	2
51	28.4	431.0999	269.0447	Apigenin 7-*O*-glucoside	1
52	28.7	431.0999	269.0447	Apigenin-hexoside	2
53	29.1	417.0843	285.0400	Kaempferol-pentoside	2
54	29.8	447.0948	285.0400	Luteolin 7-*O*-glucoside	1
55	29.9	417.0843	285.0400	Kaempferol pentoside isomer	2

^a^ MSI level of identification according to Sumner et al., 2007 [20]. ^b^ HHDP, hexahydroxydiphenoyl.

**Table 3:** Amounts of selected compounds detected in pomegranate peels M1–M14.

	Cyanidin 3-*O*-glucoside	Delphinidin 3-*O*-glucoside	Ellagic Acid	Gallic Acid	Punicalagin	Punicalin
M1	0.15 ± 0.02	nd	13.20 ± 1.89 ^a^	0.55 ± 0.10 ^a^	87.86 ± 20.16 ^a^	4.22 ± 0.11 ^a^
M2	0.13 ± 0.01	nd	7.07 ± 2.29 ^bc^	0.71 ± 0.01 ^ac^	72.91 ± 18.59 ^a^	3.43 ± 0.41 _a_
M3	0.35 ± 0.01	nd	8.19 ± 1.68 ^acd^	0.75 ± 0.02 ^ad^	93.86 ± 22.66 ^a^	3.85 ± 0.29 ^a^
M4	0.34 ± 0.01	nd	5.65 ± 1.39 ^bde^	0.65 ± 0.02 ^a^	58.16 ± 9.72 ^a^	2.27 ± 0.22 ^a^
M5	0.17 ± 0.01	nd	11.55 ± 0.69 ^af^	0.77 ± 0.04 ^a^	107.28 ± 3.86 ^ac^	3.70 ± 0.11 ^a^
M6	0.14 ± 0.01	nd	13.80 ± 2.51 ^a^	1.52 ± 0.06 ^b^	85.16 ± 11.90 ^a^	6.73 ± 0.46 ^ab^
M7	0.14 ± 0.01	nd	14.67 ± 0.92 ^a^	0.68 ± 0.01 ^ah^	142.38 ± 4.16 ^bd^	6.58 ± 0.74 ^ab^
M8	0.12 ± 0.01	nd	12.66 ± 1.76 ^a^	0.96 ± 0.01 ^bcdh^	112.79 ± 5.01 ^acd^	4.22 ± 0.45 ^a^
M9	0.47 ± 0.05	nd	7.15 ± 0.56 ^bdfg^	0.47 ± 0.01 ^afi^	85.04 ± 6.85 ^a^	4.04 ± 0.66 ^a^
M10	0.44 ± 0.03	nd	4.60 ± 0.30 ^bdh^	0.36 ± 0.01 ^afi^	89.79 ± 1.57 ^a^	3.02 ± 0.21 ^a^
M11	0.13 ± 0.01	nd	11.96 ± 0.68 ^acg^	0.20 ± 0.03 ^bdgij^	83.35 ± 6.90 ^a^	3.43 ± 0.61 ^a^
M12	0.11 ± 0.01	nd	8.64 ± 0.54 ^aceghi^	0.41 ± 0.01 ^af^	80.65 ± 2.42 ^a^	3.30 ± 0.27 ^a^
M13	1.99 ± 0.05	0.52 ± 0.08	4.62 ± 0.1 ^bdi^	0.33 ± 0.01 ^ae^	52.38 ± 5.99 ^a^	2.36 ± 0.23 ^a^
M14	0.61 ± 0.07	nd	5.87 ± 0.40 ^bdi^	0.56 ± 0.01 ^ac^	59.32 ± 5.37 ^a^	2.50 ± 0.27 ^a^

Data are expressed as mg of compounds in g of dried hydroalcoholic extract ± standard deviation. Different letters within each column indicate statistically significant differences at *p* ≤ 0.05 in an ANOVA + Tukey’s pairwise *t*-test analysis.

**Table 4:** Antioxidant activities of pomegranate peels M1-M14. Different letters within each column indicate statistically significant differences at *p* ≤ 0.05 in an ANOVA and Tukey’s pairwise *t*-test analysis.

Sample	DPPH TEAC mM	ABTS TEAC mM
M1	45.8 ± 4.1 ^a^	7.4 ± 0.2 ^a^
M2	53.3 ± 5.3 ^ac^	8.4 ± 0.1 ^b^
M3	72.8 ± 6.1 ^b^	10.3 ± 0.1 ^bc^
M4	49.7 ± 6.1 ^ad^	10.2 ± 0.1 ^bc^
M5	67.4 ± 4.5 ^a^	7.6 ± 0.2 ^a^
M6	45.3 ± 3.6 ^a^	8.5 ± 0.1 ^b^
M7	63.1 ± 3.9 ^bc^	10.2 ± 0.1 ^bc^
M8	59.6 ± 5.5 ^bcd^	8.4 ± 0.1 ^b^
M9	60.6 ± 5.4 ^bcd^	9.4 ± 0.2 ^bcd^
M10	59.2 ± 5.1 ^bcde^	10.2 ± 0.1 ^bc^
M11	48.3 ± 2.3 ^ae^	9.3 ± 0.2 ^bcd^
M12	47.3 ± 6.8 ^a^	8.2 ± 0.2 ^b^
M13	40.1 ± 5.9 ^a^	9.4 ± 0.1 ^bcd^
M14	70.0 ± 9.1 ^b^	13.3 ± 0.2 ^bcde^

Values are expressed as means ± standard deviation (n = 3). TEAC (Trolox equivalent antioxidant capacity) is expressed as mM Trolox equivalent. DPPH: 2,2-diphenyl-1-picrylhydrazyl. ABTS: 2,20-azino-bis (3-ethylbenzothiazoline-6-sulphonic acid). Different letters within each column indicate statistically significant differences at *p* ≤ 0.05 in an ANOVA + Tukey’s pairwise *t*-test analysis.

**Table 5:** Pearson correlation coefficients (ρs) between metabolites and (**A**) antioxidants and (**B**) anti-inflammatory activities of pomegranate peel extracts. Only negative statistically significant correlations (*p* ≤ 0.260; *: *p* ≤ 0.05; **: 0.01 ≤ *p* ≤ 0.05; ***: *p* ≤ 0.001) were reported. Blue color intensity is directly proportional to *Pearson* coefficient significance.

**(A)**											
				**ABTS TEAC**				**DPPH Assay**			
				*ρ*							
Granatin				−0.485 ***				−0.557 ***			
Trisgalloyl iso 2				−0.469 ***				−0.491 ***			
HHDP iso 2				−0.464 ***				0.180			
Casuarinin				−0.441 **				−0.137			
Digalloyl HHDP iso 3				−0.364 **				−0.160			
Galloyl-hexoside iso 1				−0.351 **				0.260			
Ellagic acid				−0.319 *				−0.436 **			
Pedunculagin				−0.313 *				0.103			
Galloyl-hexoside iso 3				−0.312 *				−0.084			
HHDP iso 1				−0.303 *				−0.035			
Digalloyl iso 3				−0.288 *				0.243			
Pedunculagin iso 2				−0.269 *				0.373			
Galloyl-hexoside iso 3				−0.260				−0.141			
Galloyl-hexoside iso 3				−0.260				−0.141			
**(B)**											
			**IL-1β**			**IL-6**			**TNF-α**		
						*ρ*					
Galloyl-hexoside iso 2			−0.900 ***			0.022			−0.374 **		
Pedunculagin			−0.728 ***			−0.057			−.591 ***		
HHDP iso 2			−0.677 ***			0.083			−0.323 *		
Pedunculagin iso 2			−0.673 ***			0.042			−0.310 *		
Punicalagin iso 2			−0.600 ***			−0.060			−0.634 ***		
Pedunculagin iso 1			−0.588 ***			−0.096			−0.295 *		
Trisgalloyl iso 1			−0.582 ***			−0.085			−0.460 ***		
Galloyl-gluconate iso 2			−0.555 ***			−0.222			−0.359 **		
Galloylpunicalin			−0.544 ***			−0.092			−0.576 ***		
Digalloyl			−0.520 ***			−0.112			−0.436 **		
Digalloyl HHDP iso 2			−0.518 ***			0.131			−0.273 *		
Castalagin			−0.515 ***			−0.127			−0.615 ***		
Casuarinin			−0.499 ***			−0.248			−0.454 ***		
Punicalagin iso 1			−0.489 ***			−0.241			−0.703 ***		
Galloyl-gluconate iso 1			−0.446 **			−0.296 *			−0.382 **		
Ellagic acid deoxy			−0.438 **			−0.361 **			−0.545 ***		
Ellagic acid pentoside			−0.429 **			−0.356 **			−0.504 ***		
Digalloyl gallagyl iso 1			−0.416 **			−0.381 **			−0.109		
Digalloyl gallagyl iso 2			−0.410 **			−0.402 **			−0.108		
Digalloyl HHDP iso 3			−0.403 **			−0.500 ***			−0.221		
Casuariin			−0.395 **			−0.298 *			−0.133		
Galloyl-hexoside iso 3			−0.268 *			−0.470 ***			−0.038		
Ellagic acid hexoside			−0.217			−0.329 *			−0.485 ***		
Digalloyl iso 2			−0.127			−0.376 **			−0.376 **		

## Data Availability

Data are contained within the article and supplementary material.

## Supplementary Materials

The following supporting information can be downloaded at: https://www.mdpi.com/article/10.3390/antiox11112262/s1, Figure S1: UHPLC-HR-ESI-MS profiles of the M5 sample in the negative ion mode; Figure S2: Heatmap visualization of 45 phenolic compounds detected and quantified in the peel samples; Figure S3. MRM chromatogram acquired in the negative ion mode of standards (A) and *P. granatum* peel extract (B). Figure S4. MRM chromatogram acquired in the positive ion mode of standards (A) and *P. granatum* peel extract (B). Figure S5: Heatmap visualization of antioxidant (A) and anti-inflammatory (B) activities of peel samples; Figure S6: Principal component analysis (PCA) according the genotypes of the peel samples. Figure S7. PCA loadings plots of components 1 and 2 (PC1 and PC2), respectively, of chemical data performed on the 14 peel samples. Figure S8. Loadings of PCA from Figures S4. Table S1. Loading (A) and Score (B) plots of chemical data of pomegranate varieties under study from Figure 4. Table S2. Loading (A) and Score (B) plots of chemical data of pomegranate varieties under study from Figure S4. Table S3. List of Pearson correlation coefficients (rs) between each metabolite under study and all the analyzed bioactivities related to antioxidant (ABTS, TEAC, and DPPH) and anti-inflammatory (IL-β, IL-6, and TNF- α) activities.
