# Supplementary material for: Comparative Chemical Analysis of Eight Punica granatum L. Peel Cultivars and Their Antioxidant and Anti-Inflammatory Activities
Source: Antioxidants (Basel). 2022 Nov 16;11(11):2262. doi: 10.3390/antiox11112262 (PMC9687128; doi:10.3390/antiox11112262)
Supplement: Supplementary file 1 [file antioxidants-11-02262-s001.zip › antioxidants-1969108-supplementary_def_11.pdf]

Supplementary Materials

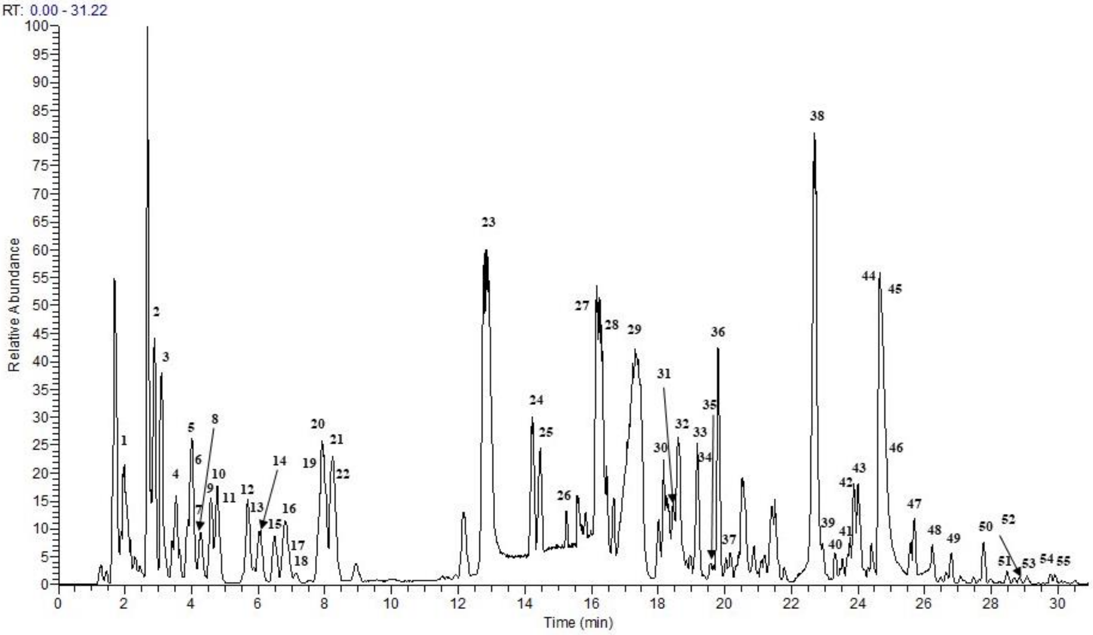

Figure S1. UHPLC-HR-ESI-MS profiles of the M5 sample in negative ion mode.

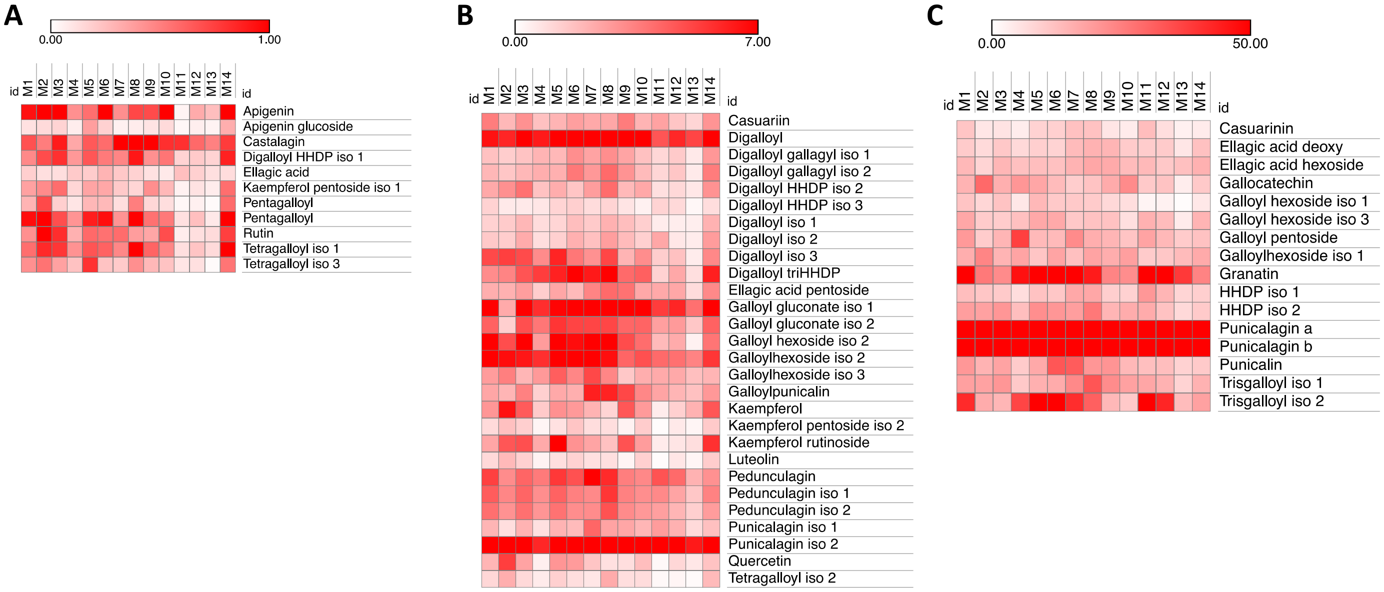

Figure S2. Heatmap visualization of 45 phenolic compounds detected and quantified in the peel samples classified in «low-» «medium-» and «high-abundant» compounds (**A, B** and **C**, respectively). Increasing red color shades are directly proportional to compound intensity. Data represent, for each, LC-MS signal intensities and are expressed as average values/100,000,000.

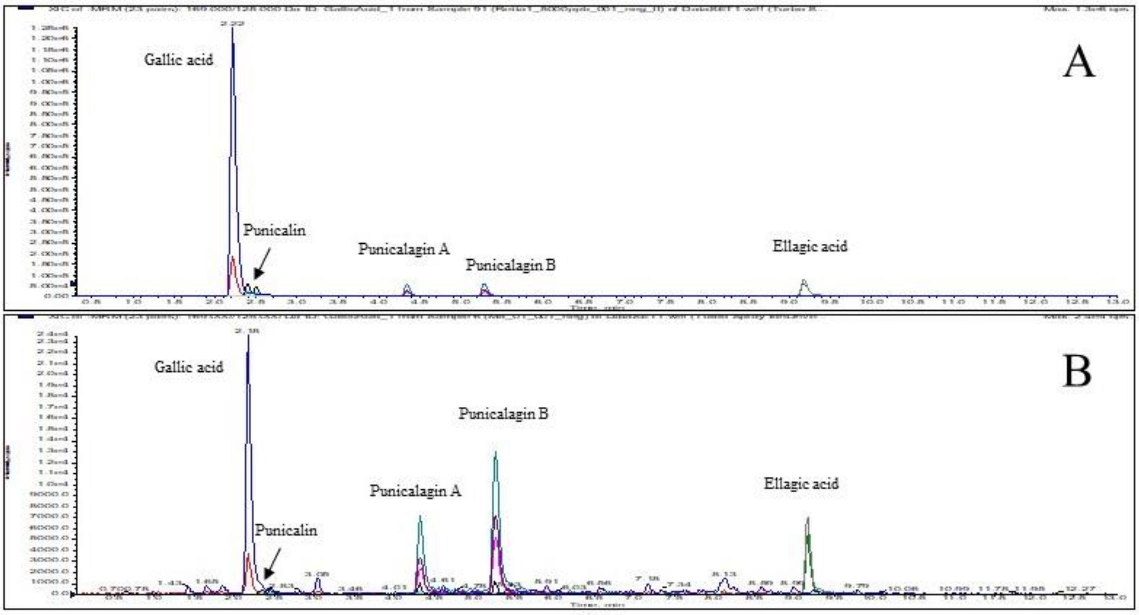

Figure S3. MRM chromatogram acquired in negative ion mode of standards (**A**) and *P. granatum* peel extract (**B**).

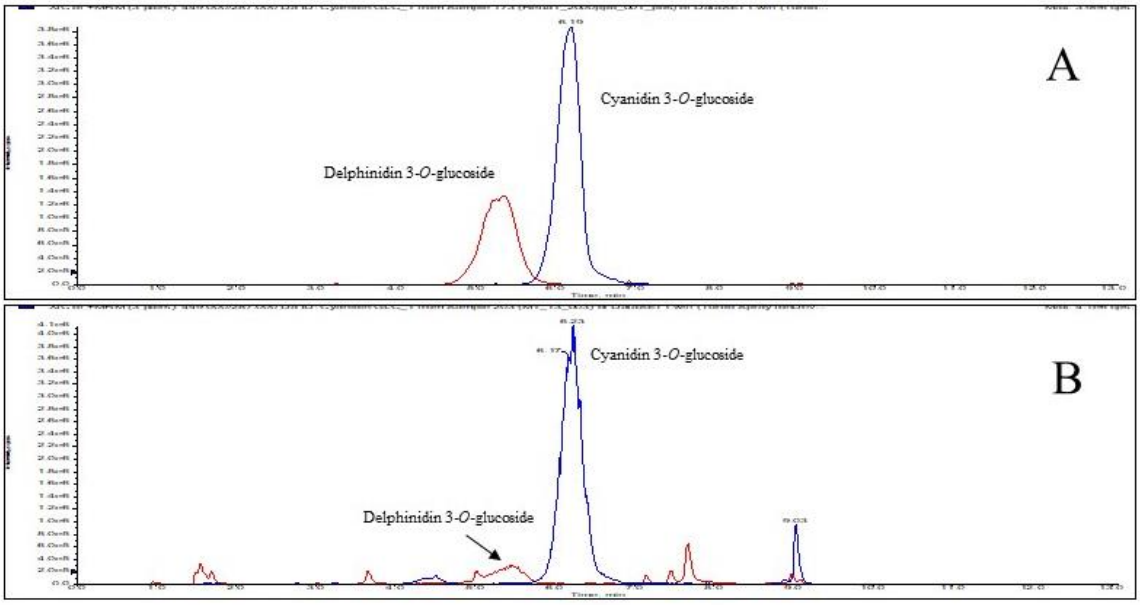

Figure S4. MRM chromatogram acquired in positive ion mode of standards (**A**) and *P. granatum* peel extract (**B**).

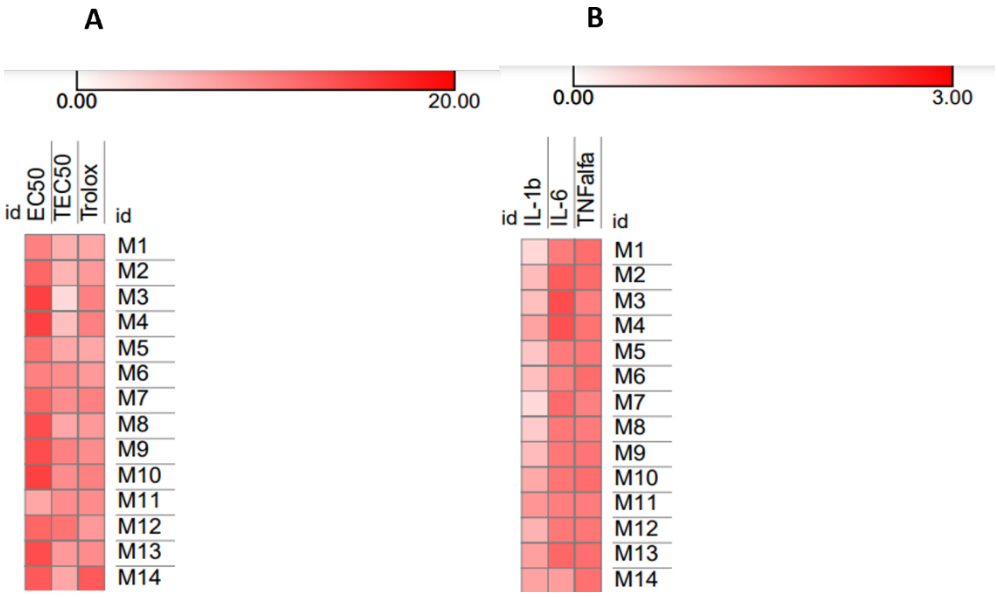

Figure S5. Heatmap visualization of antioxidant (**A**) and anti-inflammatory (**B**) activities of the peel samples. Increasing red color shades are inversely proportional to the bioactivities under study. Data are expressed as average values.

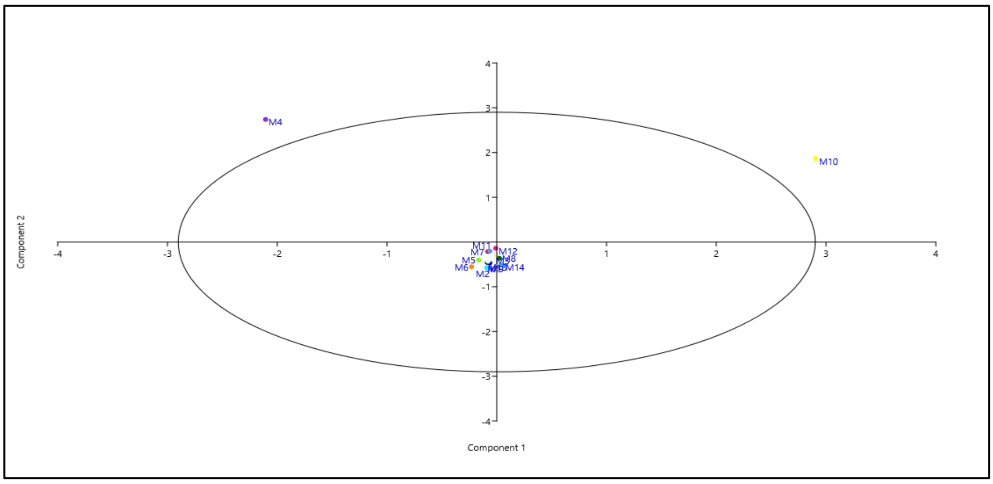

Figure S6. Principal Component Analysis (PCA) according the genotypes of the peel samples. Component 1 and 2 explained, respectively, 48.7% and 32.5% of the total variance.

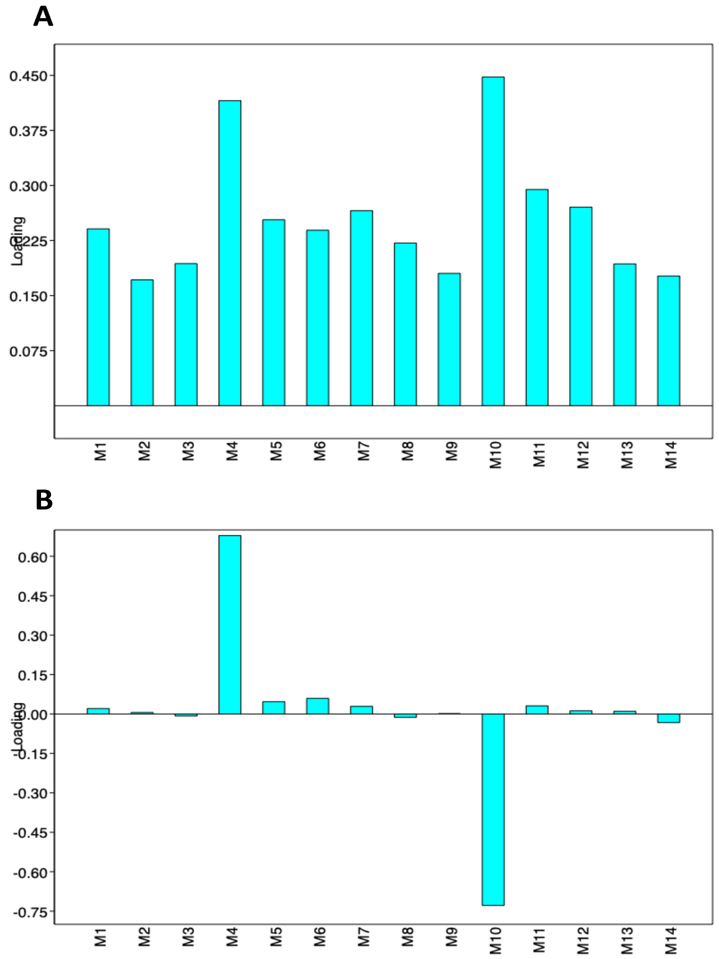

Figure S7. PCA loadings plots of component 1 and 2 (PC1 and PC2), respectively, of chemical data performed on the 14 peel samples.

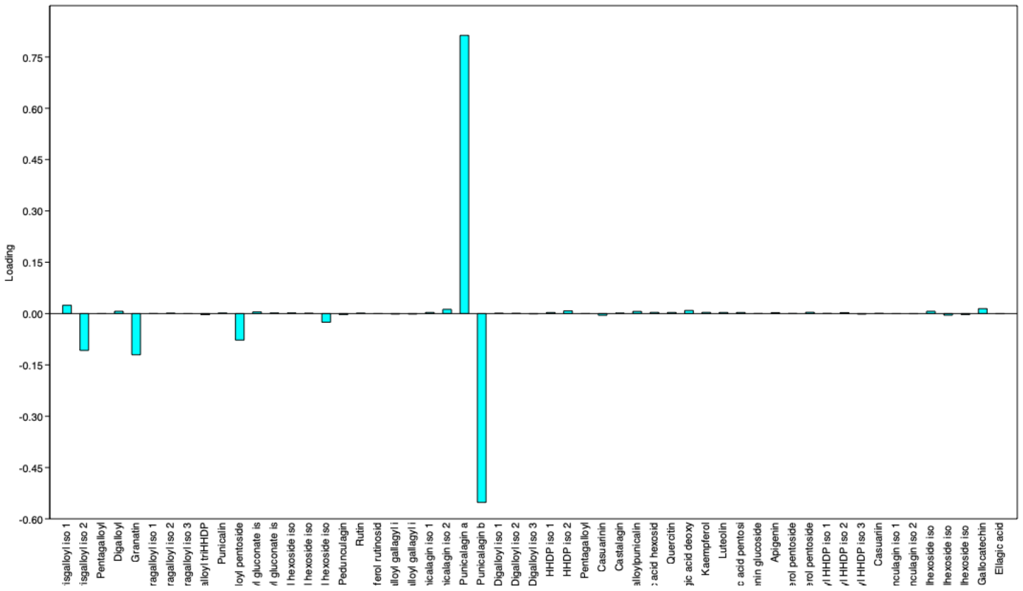

Figure S8. Loadings of PCA from Figures S4

Table S1. Loadings (A) and Scores (B) plots of chemical data of pomegranate varieties under study from Figure 4.

A. LOADINGS														
	**PC 1**	**PC 2**	**PC 3**	**PC 4**	**PC 5**	**PC 6**	**PC 7**	**PC 8**	**PC 9**	**PC 10**	**PC 11**	**PC 12**	**PC 13**	**PC 14**
**M1**	0.24089	0.020598	0.25666	0.040637	0.10267	0.021214	0.053307	−0.034863	0.30565	0.19731	−0.77753	−0.0057932	−0.3062	0.16816
**M2**	0.17151	0.0055384	−0.061507	0.35793	0.59302	0.46182	0.34775	−0.25837	−0.15164	−0.077399	0.069625	0.21799	0.03907	−0.056116
**M3**	0.19359	−0.007772	−0.11983	0.37561	−0.04144	−0.028913	−0.079429	−0.13762	0.32506	0.2573	0.11131	−0.37073	0.5315	0.41836
**M4**	0.41555	0.6784	−0.52831	−0.2244	0.033182	−0.11378	−0.058651	−0.069256	−0.032777	−0.070699	−0.075976	0.0020124	−0.015834	−0.056971
**M5**	0.25329	0.046767	0.3093	−0.080038	0.40939	−0.14788	−0.42817	0.021967	0.36562	0.2312	0.42593	0.10599	−0.18444	−0.21097
**M6**	0.23901	0.059234	0.32056	0.10431	0.27111	−0.52503	0.23738	0.2843	−0.4818	−0.016744	−0.036324	−0.29576	0.13345	−0.019494
**M7**	0.26574	0.028845	0.24007	0.13035	−0.39155	−0.34952	0.34693	−0.31929	0.18242	−0.12774	0.1135	0.5204	0.13232	−0.073146
**M8**	0.2216	−0.012663	0.071601	0.43512	−0.29205	0.0772	−0.53632	−0.27227	−0.49572	0.06302	−0.04248	0.02011	−0.225	−0.026534
**M9**	0.18026	0.0015036	−0.08242	0.43417	−0.19904	0.088267	0.15779	0.33789	0.32284	−0.39296	0.1329	−0.33841	−0.35056	−0.26835
**M10**	0.44772	−0.72821	−0.40721	−0.23441	0.066677	−0.15082	−0.022392	−0.080103	−0.028716	−0.062566	−0.053075	−0.024434	−0.026595	−0.074262
**M11**	0.29455	0.030869	0.3901	−0.34096	−0.17374	0.4416	−0.047306	−0.10252	−0.01869	−0.15422	−0.088485	−0.2779	0.40041	−0.36932
**M12**	0.2705	0.012137	0.21202	−0.24416	−0.043974	0.20655	−0.0049317	0.12811	−0.078897	−0.34844	0.25311	0.053232	−0.20645	0.72383
**M13**	0.19317	0.010339	−0.029232	−0.10744	−0.276	0.22668	0.39374	0.14254	−0.14071	0.70805	0.24922	−0.062948	−0.24318	−0.030413
**M14**	0.17663	−0.032727	−0.071331	0.15596	−0.053894	0.16508	−0.19003	0.69504	−0.024421	0.070667	−0.14329	0.49592	0.34106	−0.024378

B. SCORES														
	**PC 1**	**PC 2**	**PC 3**	**PC 4**	**PC 5**	**PC 6**	**PC 7**	**PC 8**	**PC 9**	**PC 10**	**PC 11**	**PC 12**	**PC 13**	**PC 14**
**Trisgalloyl iso 1**	0.45723	−0.17837	0.45525	33.343	−17.088	16.741	−18.578	−13.759	−13.347	−14.482	−0.17457	−0.20539	−22.169	0.6202
**Trisgalloyl iso 2**	12.639	0.88942	28.888	−0.63191	21.699	−23.303	−30.023	13.636	−19.115	−1.848	0.13708	0.70367	0.22554	−0.27275
**Pentagalloyl**	−0.51409	−0.048053	−0.25318	−0.79551	0.0019487	0.065507	0.19087	−0.20081	−0.40106	−0.13375	−0.022218	−0.39366	−0.57042	−0.070247

**Digalloyl**	−0.10536	−0.072245	−0.10255	0.86895	−0.93914	0.0074129	−0.35707	0.92505	0.38465	0.24525	14.946	13.468	−0.81781	0.56591
**Granatin**	24.424	1.056	53.552	−19.198	−0.63713	14.882	17.391	−0.6177	0.94978	11.063	0.51835	−0.28801	−1.007	0.32338
**Tetragalloyl iso 1**	−0.49387	−0.048576	−0.25223	−0.67314	−0.056993	0.056302	−0.031405	0.010046	−0.4366	−0.013984	−0.057814	−0.10542	−0.19536	0.055953
**Tetragalloyl iso 2**	−0.47351	−0.056763	−0.24461	−0.51499	0.15981	0.20113	−0.11394	−0.15481	−0.57779	0.044236	−0.13692	0.35431	−0.59813	−0.37409
**Tetragalloyl iso 3**	−0.50898	−0.046272	−0.24726	−0.81007	0.023311	0.015249	0.092627	−0.19769	−0.14265	−0.059348	0.11601	−0.48685	−0.71878	−0.15219
**Digalloyl triHHDP**	−0.26777	−0.0062353	−0.13793	0.29616	−0.0503	−12.268	−12.661	0.36481	−19.181	0.134	0.69396	18.985	0.25766	−10.832
**Punicalin**	0.42384	−0.01948	0.89486	2.885	−0.39591	−41.421	40.923	0.22798	−0.91046	−10.179	0.048993	−0.91724	0.7917	−0.3855
**Galloyl pentoside**	0.43866	0.66212	−0.19607	0.068009	−14.166	−0.302	0.59299	−10.336	22.536	−0.72085	−42.764	19.653	0.44775	−0.91013
**Galloyl gluconate iso 1**	−0.13987	−0.057947	−0.035254	0.47107	−10.072	−0.59168	−0.31912	18.531	1.074	−0.44284	0.083697	−2.26	−10.421	11.276
**Galloyl gluconate iso 2**	−0.3065	−0.042541	−0.11713	−0.23662	−0.2243	−0.77536	−0.54691	0.48302	0.4521	0.3527	0.17754	−0.52305	0.17231	0.28371
**Galloyl hexoside iso 1**	−0.12126	−0.044697	0.15203	11.473	12.951	−18.101	−0.93997	−11.507	26.835	18.545	−0.42728	−0.23646	−11.628	11.834
**Galloyl hexoside iso 2**	−0.23474	−0.046563	0.092562	0.68543	0.078782	−13.681	−0.84233	−14.906	12.363	0.97444	−0.28714	22.823	−0.85495	0.84871
**Galloyl hexoside iso 3**	0.14641	0.17664	0.52683	0.54602	11.673	0.69307	−11.991	34.132	10.837	−0.76407	−28.033	−0.39297	17.945	10.079

**Pedunculagin**	−0.28559	−0.011488	0.013801	−0.27352	−0.36548	−0.25713	0.059208	−0.73392	0.22824	−0.056314	0.17855	0.80241	−0.22153	0.49501
**Rutin**	−0.49932	−0.056365	−0.26054	−0.74179	0.098976	0.030685	0.33685	−0.25888	−0.2192	−0.06325	0.14064	−0.30853	0.085281	0.11515
**Kaempferol rutinoside**	−0.35823	−0.043546	−0.18858	−0.047208	1.059	−0.10063	−0.55265	12.871	22.065	10.462	24.471	11.436	0.83492	−32.441
**Digalloyl gallagyl iso 1**	−0.41791	−0.030722	−0.19838	−0.45602	−0.15694	−0.29494	−0.0074695	0.39748	−0.66374	−0.46881	0.021973	−0.04896	−0.45254	−0.4623
**Digalloyl gallagyl iso 2**	−0.40158	−0.03094	−0.18663	−0.31536	−0.20331	−0.34667	−0.18594	0.57585	−0.90552	−0.48886	−0.12093	0.16611	−0.39947	−0.52446
**Punicalagin iso 1**	−0.41292	−0.065405	−0.12012	−0.59479	−0.54021	−0.076848	0.29658	−0.30519	0.24225	−0.60082	0.42805	−0.05812	0.17379	−0.16279
**Punicalagin iso 2**	0.038372	−0.11109	0.17694	13.101	−16.473	0.025595	0.71728	0.085639	12.141	−11.654	19.951	−0.0052606	15.153	0.76666
**Punicalagin a**	47.058	−52.379	−14.727	−1.022	−0.059481	−0.37741	−0.14934	0.0062091	−0.047697	0.0059639	−0.088062	0.043663	0.084069	0.017499
**Punicalagin b**	40.866	49.016	−32.841	−0.47993	−0.24404	−0.19048	−0.05275	−0.13668	−0.24609	0.13714	0.57178	−0.3245	0.068397	0.1817
**Digalloyl iso 1**	−0.45509	−0.053399	−0.23804	−0.47957	−0.08092	−0.079323	0.083684	0.12327	−0.32842	−0.034595	−0.082005	−0.5166	0.26617	−0.0091945
**Digalloyl iso 2**	−0.43777	−0.050879	−0.18077	−0.49889	−0.22801	0.1522	−0.062474	0.18059	−0.45019	−0.13641	−0.31768	−0.95949	21.604	−1.209
**Digalloyl iso 3**	−0.3391	−0.030804	−0.14724	−0.066236	0.85463	−0.070633	−0.66261	−0.61594	0.30187	11.012	−0.46306	0.47129	−0.1285	1.425
**HHDP iso 1**	0.13569	−0.037438	0.62557	0.45949	−12.876	23.817	−0.092139	−11.872	−0.93745	−0.69159	0.90335	10.898	38.171	0.77034

**HHDP iso 2**	0.40207	−0.048641	0.54998	23.356	0.99748	0.028951	−19.874	−23.699	−0.63645	24.098	0.52071	−2.847	17.754	−0.41495
**Pentagalloyl**	−0.48678	−0.048859	−0.24515	−0.60904	0.25825	0.22663	0.19688	0.03571	−0.51016	−0.033545	−0.23917	0.47398	−0.01011	−0.13182
**Casuarinin**	−0.12239	0.0058099	0.48606	0.020676	−0.77847	0.24632	−0.41082	−16.386	−0.48536	−0.26929	−24.015	−0.35415	0.63454	−34.146
**Castalagin**	−0.48024	−0.056718	−0.22051	−0.66699	−0.36388	−0.0042659	0.17369	−0.33925	−0.22913	−0.33335	0.25136	−0.46829	−0.84655	−0.47304
**Galloylpunicalin**	−0.35933	−0.087444	−0.14142	−0.03876	−0.96559	−0.058065	0.068543	−0.49106	0.012133	−0.69041	0.73067	0.46167	−11.998	−15.151
**Ellagic acid hexoside**	0.21419	−0.022691	0.11739	1.19	−16.157	11.136	−0.32779	25.617	0.95428	24.487	0.38952	−0.82526	−0.46867	−21.378
**Quercitin**	−0.43569	−0.071207	−0.19053	−0.3977	0.96661	0.16947	0.78997	−0.36518	−0.12178	0.41244	0.58686	0.43052	0.63442	13.538
**Ellagic acid deoxy**	0.036765	−0.082792	0.10961	11.081	−13.648	0.82605	−0.82467	1.504	0.94411	−18.312	0.464	11.284	−0.54633	1.174
**Kaempferol**	−0.38091	−0.073165	−0.25742	0.015498	0.76122	0.64694	11.285	0.87865	0.81122	0.5015	0.62752	0.23463	10.639	0.39821
**Luteolin**	−0.47872	−0.069534	−0.2433	−0.66717	0.21177	0.03206	0.33039	−0.1531	−0.40468	0.0578	−0.069105	0.036378	0.34408	1.284
**Ellagic acid pentoside**	−0.39042	−0.06515	−0.16293	−0.30562	−0.41429	0.27651	−0.12387	0.16745	0.027532	−0.77716	0.41093	0.021291	−0.63945	−0.12891
**Apigenin glucoside**	−0.51889	−0.048998	−0.25441	−0.87038	−0.044649	−0.027223	0.18616	−0.16172	−0.21531	−0.11012	0.15444	−0.70332	−0.56886	−0.027306
**Apigenin**	−0.48324	−0.066022	−0.26293	−0.64995	0.18217	−0.0054884	0.36507	−0.035047	−0.37565	0.018196	−0.18548	−0.71324	0.35506	0.8014

**Kaempferol pentoside iso 1**	−0.51092	−0.049966	−0.25684	−0.80057	−0.028148	−0.0030078	0.25137	−0.10902	−0.12441	−0.12768	0.044057	−0.76361	−0.30857	0.19073
**Kaempferol pentoside iso 2**	−0.47749	−0.072932	−0.24054	−0.66224	0.18888	−0.082584	0.35121	−0.082317	−0.48836	0.074349	−0.096489	−0.10103	0.50349	11.228
**Digalloyl HHDP iso 1**	−0.49823	−0.05024	−0.25369	−0.74128	−0.040259	0.0016013	0.089577	−0.18206	−0.29812	−0.010543	0.09089	−0.4926	−0.225	0.13766
**Digalloyl HHDP iso 2**	−0.40593	−0.062018	−0.23668	−0.2184	−0.019762	0.1243	−0.25469	−0.40331	−0.095845	0.4194	−0.14244	0.46756	11.731	14.456
**Digalloyl HHDP iso 3**	−0.47835	−0.033785	−0.1849	−0.78866	−0.069075	−0.0027778	0.096373	−0.091523	−0.33228	−0.29466	−0.13144	−0.73698	−0.59539	0.21848
**Casuariin**	−0.39715	−0.046895	−0.16625	−0.40734	−0.15163	0.17643	0.27289	0.42942	0.42986	−0.16717	−0.8997	−14.746	0.10608	−0.16713
**Pedunculagin iso 1**	−0.33485	−0.033642	−0.12863	−0.20911	0.012351	0.21093	−0.50494	−0.14507	−0.065754	0.4157	−0.51653	−0.90081	−0.46394	0.3
**Pedunculagin iso 2**	−0.3557	−0.033024	−0.15275	−0.26708	0.017929	0.066864	−0.3152	−0.28987	0.066395	0.33386	−0.34889	−0.95683	−0.60033	0.18689
**Galloylhexoside iso 1**	0.33664	−0.028673	−0.21749	19.445	16.531	20.494	2.213	17.732	−30.282	24.551	−14.601	14.086	−14.229	0.094449
**Galloylhexoside iso 2**	−0.20306	0.0064948	−0.0046329	0.14234	0.43507	−0.45301	0.44192	−0.22592	0.10571	13.247	−0.16278	19.189	10.553	0.58913
**Galloylhexoside iso 3**	−0.36943	−0.015413	−0.10368	−0.52158	0.14335	−0.19828	0.35671	−0.68951	−0.26244	0.26572	0.96489	17.423	−0.69317	−0.77014

**Gallocatechin**	0.32893	−0.05907	−0.40907	14.341	44.656	22.096	12.233	−11.717	17.529	−31.561	0.63778	−0.38119	−0.75937	−10.626
**Ellagic acid**	−0.51634	−0.043453	−0.24777	−0.88324	−0.096491	−0.021689	0.25372	−0.24327	−0.30971	−0.18307	0.080526	−0.84256	−0.60572	0.017906

Table S2. Loadings (A) and Scores (B) plots of chemical data of pomegranate varieties under study from Figure S4.

A. **LOADINGS**

	PC 1	PC 2	PC 3	PC 4	PC 5	PC 6	PC 7	PC 8	PC 9	PC 10	PC 11	PC 12	PC 13
**Trisgalloyl iso 1**	0.024202	−0.035827	−0.0034728	0.39452	−0.25724	0.43917	−0.060946	−0.22789	0.0054869	−0.095182	−0.12349	−0.33115	−0.047687
**Trisgalloyl iso 2**	−0.10763	−0.037549	0.4795	0.17284	0.28322	−0.097547	0.43593	−0.30229	−0.020384	−0.15201	0.068467	−0.042377	−0.12414
**Pentagalloyl**	−0,22811	−0.0015655	−0.0027301	0.006893	0.013317	0.010689	0.0052194	−0.017685	−0.00019983	0.021431	0.045315	−0.0020976	0.011239
**Digalloyl**	0.0066682	−0.0061503	−0.023014	0.14252	−0.12788	−0.0026705	0.11503	0.033446	0.040619	−0.13494	0.16802	−0.12947	0.077196
**Granatin**	−0.12048	−0.10478	0.83003	−0.1829	−0.07495	0.074245	−0.24499	0.14712	0.022476	0.029513	−0.024168	−0.074624	0.13158
**Tetragalloyl iso 1**	0.00014945	−0.0021792	−0.0060717	0.017755	0.00461	0.014436	0.042679	−0.026711	−0.0028931	0.034658	0.072727	0.026369	−0.005022
**Tetragalloyl iso 2**	0.0012706	−0.003834	−0.0094639	0.033067	0.032828	0.044572	0.029612	−0.041538	−0.018651	0.050237	0.11487	−0.033088	0.054023
**Tetragalloyl iso 3**	−0.00014779	−0.0010642	−0.00096957	0.0078621	0.016207	0.0090434	0.01966	0.01783	−0.0042471	0.0049029	0.02904	−0.0153	0.030567
**Digalloyl triHHDP**	−0.0030756	0.000637	−0.0012713	0.1723	−0.0099997	−0.057007	0.19701	−0.21246	−0.15257	−0.047775	0.28526	0.012105	0.10646

**Punicalin**	0.0015688	−0.052824	0.076249	0.45763	−0.097885	−0.65513	−0.39324	−0.16975	0.11387	−0.061535	−0.10425	0.12404	−0.0027695
**Galloyl pentoside**	−0.077444	0.096993	0.052055	0.12979	−0.19333	0.027953	−0.11651	0.29412	0.23646	0.48775	0.177	−0.048665	−0.09861
**Galloyl gluconate iso 1**	0.0046995	−0.0055466	−0.00095582	0.12054	−0.13575	−0.10272	0.19368	0.062039	0.20089	−0.044249	−0.28225	−0.059014	−0.05282
**Galloyl gluconate iso 2**	0.0016377	0.00061402	0.009959	0.086512	−0.025439	−0.067151	0.14773	0.078663	−0.013823	0.0032083	−0.019003	0.061254	−0.021592
**Galloyl hexoside iso 1**	0.0017405	−0.021398	0.014961	0.28968	0.1605	−0.090553	0.073673	0.43523	−0.19517	0.09558	−0.12394	−0.19101	−0.081621
**Galloyl hexoside iso 2**	0.0010581	−0.019701	0.017001	0.23511	0.0010672	−0.022719	0.032755	0.25981	−0.18924	0.07352	0.23806	−0.22038	−0.096821
**Galloyl hexoside iso 3**	−0.025228	−0.022961	0.067503	0.094887	0.16502	0.041801	0.34867	−0.029187	0.43432	0.31757	−0.12997	0.16814	−0.17105
**Pedunculagin**	−0.0027734	−0.0058872	0.027511	0.077346	−0.04443	0.01177	−0.022232	0.080398	−0.03488	−0.0067374	0.1574	−0.036324	−0.06388
**Rutin**	0.0013014	−0.00082195	−0.0048021	0.011156	0.025952	−0.0012784	−0.012112	0.0098186	−0.0021443	0.0073839	0.063526	0.071712	−0.019327
**Kaempferol rutinoside**	−0,49382	−0.009468	−0.019801	0.06028	0.1538	−0.046785	0.21037	0.29141	0.0469	−0.19341	0.18097	0.19083	0.50132
**Digalloyl gallagyl iso 1**	−0.0013705	−0.0011808	−0.00060191	0.048864	−0.012598	−0.033843	0.064988	−0.082149	0.043987	0.00022106	0.071252	−0.0020921	0.046749
**Digalloyl gallagyl iso 2**	−0.0013676	−0.0028956	−0.0027856	0.064877	−0.020284	−0.033205	0.092385	−0.121	0.045052	0.017859	0.088557	−0.0071863	0.048853
**Punicalagin iso 1**	0.0032249	−0.0049232	0.012772	0.031442	−0.063275	0.00028684	−0.013847	0.055834	0.051274	−0.060169	0.089812	0.071629	−0.0077964

**Punicalagin iso 2**	0.01236	−0.020817	0.0039655	0.18712	−0.23075	−0.0087363	−0.082022	0.11747	0.16734	−0.28851	0.054698	0.19012	−0.06778
**Punicalagin a**	0.81314	0.54449	0.20032	0.012601	−0.008251	−0.018705	0.023952	−0.0040629	−0.003154	0.012668	0.0071799	0.0090891	−0.0042243
**Punicalagin b**	−0.55194	0.81818	0.0073987	0.042602	−0.035193	0,89888	−0.0066832	−0.024209	−0.038908	−0.060588	−0.024351	0.027466	0.010665
**Digalloyl iso 1**	0.0010996	−0.002865	−0.0084352	0.037769	−0.00095782	−0.0099353	0.036588	−0.021916	0.013693	0.032365	0.02174	0.095238	−0.0096627
**Digalloyl iso 2**	0.00076331	−0.0055976	−0.0011478	0.031038	−0.019923	0.02666	0.047538	−0.046045	0.035576	0.060287	−0.0042014	0.36856	0.050774
**Digalloyl iso 3**	−0.00089434	−0.0041859	−0.0027692	0.097313	0.12117	0.058459	0.059169	0.10766	−0.12986	0.12067	0.074847	−0.04533	−0.13704
**HHDP iso 1**	0.0030505	−0.033554	0.080129	0.053975	−0.16736	0.35022	−0.14484	−0.10559	0.019196	−0.096435	0.25451	0.44784	−0.16588
**HHDP iso 2**	0.0076883	−0.023667	0.046331	0.36091	0.11173	0.23997	−0.019041	0.027348	−0.45828	0.00031029	−0.36971	0.31291	0.051133
**Pentagalloyl**	0,39601	−0.0041759	−0.008789	0.016345	0.047702	0.019608	0.013904	−0.040771	0.01919	0.066635	0.14001	0.031583	0.013223
**Casuarinin**	−0.0049086	−0.028363	0.085047	0.12447	−0.10544	0.16382	−0.085449	−0.017	−0.029534	0.2786	−0.007856	0.16121	0.21182
**Castalagin**	0.0014439	−0.0024623	−0.00010398	0.023485	−0.038298	0.014027	−0.0055263	0.00269	0.0068274	−0.027024	0.034177	−0.025499	0.059533
**Galloylpunicalin**	0.0063007	−0.0076754	−0.0038235	0.089245	−0.12664	0.033653	−0.022014	0.02226	0.030319	−0.10246	0.12247	−0.089884	0.18722
**Ellagic acid hexoside**	0.0034028	−0.0053057	−0.0067986	0.085192	−0.21445	0.032795	0.20915	0.064464	0.084982	0.13505	−0.15297	0.06643	0.51297
**Quercitin**	0.0031788	−0.0086369	−0.0087605	0.030801	0.14147	−0.017051	−0.074627	0.033398	−0.036261	−0.011508	0.1469	0.087465	−0.11775

**Ellagic acid deoxy**	0.0090655	−0.013953	−0.0009021	0.15117	−0.18518	0.12549	0.1581	0.0091934	0.32207	−0.13396	0.10885	−0.14301	−0.094771
**Kaempferol**	0.0034401	−0.010986	−0.036729	0.023402	0.11505	−0.035944	−0.033349	0.09255	0.12706	0.006027	0.095021	0.16962	0.044178
**Luteolin**	0.0031714	−0.0021778	−0.005041	0.015918	0.040817	−0.0063009	−0.0051706	−0.014314	−0.010875	0.041227	0.098131	0.060079	−0.15068
**Ellagic acid pentoside**	0.0030939	−0.0062517	−0.0033165	0.04606	−0.046603	0.049747	0.043977	−0.0016348	0.090617	−0.067523	0.070786	−0.037981	0.025188
**Apigenin glucoside**	0.00019263	−0.00044048	−0.0003367	0.0013117	0.0074027	−0.0032835	0.014624	0.0073343	−0.00064549	−0.0025567	0.011951	0.010848	0.011375
**Apigenin**	0.0026972	−0.0012788	−0.0079488	0.017225	0.036535	−0.016077	−0.0017295	−0.019757	0.0062687	0.047627	0.0089595	0.099437	−0.10245
**Kaempferol pentoside iso 1**	0.00031489	−0.0010444	−0.0030276	0.005535	0.0092456	−0.0057291	0.0094621	0.014133	0.014807	0.0094492	0.0023578	0.037111	−0.020359
**Kaempferol pentoside iso 2**	0.0036592	−0.002133	−0.0043076	0.019292	0.037208	−0.023038	0,46757	−0.026425	−0.012872	0.045202	0.084739	0.088428	−0.1392
**Digalloyl HHDP iso 1**	0.0004902	−0.00085154	−0.0034392	0.013839	0.0069252	0.0073777	0.019548	−0.0013962	−0.014115	0.011476	0.03391	0.038041	−0.014307
**Digalloyl HHDP iso 2**	0.0026869	−0.0029198	−0.014007	0.064949	0.0053852	0.052759	0.028547	0.037235	−0.058473	0.062025	0.12704	0.12647	−0.18689
**Digalloyl HHDP iso 3**	−0.0015964	−0.0027374	0.0075638	0.010941	0.0030803	0.0066867	0.022041	−0.018367	0.019543	0.01892	−0.0014281	−0.0046285	−0.030532
**Casuariin**	0.00065116	−0.0052438	−0.001993	0.030328	−0.0098148	−0.00041833	0.029539	0.044474	0.11873	0.11504	−0.12288	0.10128	0.00041379
**Pedunculagin iso 1**	−0.00035177	−0.0037559	0.002143	0.064291	0.0095219	0.069647	0.063526	0.019815	−0.033127	0.09075	−0.069312	−0.0024498	−0.019745

**Pedunculagin iso 2**	−0.00051362	−0.0022549	0.0013244	0.063831	0.010006	0.047741	0.038993	0.04232	−0.033765	0.064734	−0.069201	−0.011559	−0.0069662
**Galloylhexoside iso 1**	0.0066874	0.015862	−0.068278	0.11748	0.22423	0.024809	−0.19382	−0.44614	0.02765	0.41686	0.12394	−0.17227	0.28439
**Galloylhexoside iso 2**	−0.0045993	−0.0059427	0.012097	0.10397	0.061481	−0.067904	−0.018292	0.077529	−0.09775	0.13303	0.26885	0.07859	−0.034179
**Galloylhexoside iso 3**	−0.0028581	−0.0020297	0.015402	0.04408	0.028031	−0.012672	−0.040992	0.035248	−0.083834	−0.058858	0.29686	−0.073345	0.1337
**Gallocatechin**	0.01437	0.052284	−0.04814	0.18579	0.60302	0.27525	−0.3293	0.12643	0.39556	−0.24685	−0.046072	−0.069143	0.083372
**Ellagic acid**	−0.00048393	−0.00010772	0.001435	0.00074454	0.00021949	−0.0019586	−0.00010911	−0.0044503	−0.00036756	0.0010828	−0.0016907	0.010346	−0.00023982

B. SCORES

	**PC 1**	**PC 2**	**PC 3**	**PC 4**	**PC 5**	**PC 6**	**PC 7**	**PC 8**	**PC 9**	**PC 10**	**PC 11**	**PC 12**	**PC 13**
**M1**	−4.80E+12	−2.12E+13	2.14E+13	2.66E+12	2.73E+12	5.03E+11	−9.94E+11	3.42E+11	1.62E+12	6.25E+11	−1.21E+12	−1.45E+11	−5.54E+11
**M2**	−5.20E+12	−2.66E+13	−3.68E+13	4.28E+11	1.61E+13	5.29E+12	−8.84E+12	−1.60E+12	3.83E+11	−3.75E+11	1.62E+12	6.58E+10	2.06E+10
**M3**	−5.30E+11	−1.35E+13	−3.63E+13	7.02E+12	−1.43E+12	9.22E+10	3.30E+11	4.76E+12	−2.79E+12	−6.13E+11	−2.00E+12	1.92E+12	−1.80E+12
**M4**	−1.19E+14	1.27E+14	−7.54E+12	3.93E+11	8.95E07	−1.33E+11	3.19E+11	−3.03E+11	1.05E+11	3.27E+11	3.19E+10	−3.20E+10	1.03E+11
**M5**	−9.36E+12	−1.86E+13	3.26E+12	2.62E08	1.11E+13	4.42E+11	7.08E+12	5.14E+12	−2.94E+12	−2.68E+12	4.54E+11	−5.48E+10	1.17E+12
**M6**	−1.31E09	−2.56E+13	2.81E+13	8.61E+12	6.65E+12	−1.16E+13	1.29E+11	−6.20E+12	−5.30E+11	1.75E+11	−1.58E+12	7.64E+11	−6.88E+10
**M7**	−4.57E+12	−9.88E+11	2.68E+13	1.24E+13	−1.12E+13	−5.14E+12	−6.41E+12	3.01E+12	4.03E+11	−8.16E+11	3.63E+12	1.71E+11	8.31E+10
**M8**	1.35E+12	−1.69E+13	−6.90E+11	1.85E+13	−8.61E+12	8.86E+12	2.73E+12	−4.89E+12	−4.55E+12	2.75E+11	−1.36E+11	−8.73E+11	1.98E+11
**M9**	−3.66E+12	−2.18E+13	−3.70E+13	5.75E+12	−5.67E+12	−1.41E+12	−1.21E+11	1.05E+12	6.63E+12	−2.44E08	−2.57E+12	−8.62E+11	1.23E+12
**M10**	1.65E+14	8.66E+13	−9.14E+11	6.48E+11	1.76E+12	−9.84E+11	−1.49E+10	−2.02E+11	−4.83E+10	1.60E+11	−1.19E+10	−3.07E+10	1.84E+11
**M11**	−3.55E+12	−9.45E+12	5.25E+13	−1.28E+13	−4.08E+12	8.30E+12	−1.79E+12	−7.67E+11	2.12E+12	3.40E+11	−1.14E+12	2.05E+12	8.51E+11
**M12**	−5.42E+11	−6.48E+12	2.49E+13	−1.37E+13	−6.85E+11	1.72E+12	7.55E+11	−1.82E+12	1.94E+11	−3.26E+12	3.06E+11	−1.43E+12	−2.42E+12

**M13**	−4.53E+12	−2.00E+12	−2.62E+13	−2.58E+13	−6.69E+12	−4.85E+12	−2.55E+11	2.56E+09	−5.56E+12	5.38E+11	−4.85E+11	−6.56E+11	8.95E+11
**M14**	3.08E+12	−2.39E+13	−3.47E+13	−6.74E08	−8.11E+11	−1.11E+12	9.51E+12	−1.59E+12	3.22E+12	2.12E+12	3.21E+12	9.18E+11	−6.70E+10

Table S3. List of Pearson correlation coefficients (rs) between each metabolite under study and all the analyzed bioactivities related to antioxidant (ABTS, TEAC, and DPPH) and anti−inflammatory (IL−β, IL−6, and TNF−α) activities.

	ABTS /TEAC	DPPH assay	IL−1β	IL−6	TNF−α
	ρ				
**Trisgalloyl iso 1**	−0.16	0.49	−0.58	−0.09	−0.46
**Trisgalloyl iso 2**	−0.47	−0.49	−0.23	−0.25	−0.20
**Pentagalloyl**	0.07	0.31	−0.26	−0.21	0.30
**Digalloyl**	0.28	0.75	−0.52	−0.11	−0.44
**Granatin**	−0.48	−0.56	−0.16	−0.24	−0.25
**Tetragalloyl iso 1**	0.36	0.62	−0.25	−0.33	0.00
**Tetragalloyl iso 2**	0.05	0.44	−0.35	−0.16	0.15
**Tetragalloyl iso 3**	−0.10	0.25	−0.39	−0.17	0.09
**Digalloyl triHHDP**	0.09	0.40	−0.48	−0.16	−0.18
**Punicalin**	−0.26	0.11	−0.75	−0.05	−0.19
**Galloyl pentoside**	0.06	−0.08	−0.02	0.38	−0.25
**Galloyl gluconate iso 1**	0.17	0.52	−0.45	−0.30	−0.38
**Galloyl gluconate iso 2**	0.06	0.41	−0.56	−0.22	−0.36
**Galloyl hexoside iso 1**	−0.35	0.26	−0.84	0.09	−0.17
**Galloyl hexoside iso 2**	−0.26	0.35	−0.90	0.02	−0.37
**Galloyl hexoside iso 3**	−0.26	−0.14	−0.27	−0.47	−0.04
**Pedunculagin**	−0.31	0.10	−0.73	−0.06	−0.59
**Rutin**	0.17	0.47	−0.28	0.19	0.24
**Kaempferol rutinoside**	0.11	0.36	−0.24	−0.10	0.06
**Digalloyl gallagyl iso 1**	0.21	0.42	−0.42	−0.38	−0.11
**Digalloyl gallagyl iso 2**	0.22	0.45	−0.41	−0.40	−0.11

**Punicalagin iso 1**	0.01	0.35	−0.49	−0.24	−0.70
**Punicalagin iso 2**	0.06	0.59	−0.60	−0.06	−0.63
**Punicalagin a**	0.12	0.13	0.17	−0.11	0.15
**Punicalagin b**	0.13	−0.16	0.27	0.47	−0.03
**Digalloyl iso 1**	0.32	0.73	−0.44	−0.16	−0.09
**Digalloyl iso 2**	0.35	0.61	−0.13	−0.38	−0.38
**Digalloyl iso 3**	−0.29	0.24	−0.62	0.13	0.01
**HHDP iso 1**	−0.30	−0.03	−0.25	−0.25	−0.59
**HHDP iso 2**	−0.46	0.18	−0.68	0.08	−0.32
**Pentagalloyl**	0.14	0.32	−0.20	−0.20	0.38
**Casuarinin**	−0.44	−0.14	−0.50	−0.25	−0.45
**Castalagin**	0.08	0.53	−0.51	−0.13	−0.61
**Galloylpunicalin**	0.11	0.58	−0.54	−0.09	−0.58
**Ellagic acid hexoside**	0.31	0.52	−0.22	−0.33	−0.49
**Quercitin**	−0.23	0.08	−0.37	0.18	0.36
**Ellagic acid deoxy**	0.16	0.64	−0.44	−0.36	−0.54
**Kaempferol**	0.20	0.42	−0.16	0.09	0.35
**Luteolin**	0.09	0.38	−0.33	−0.03	0.33
**Ellagic acid pentoside**	0.16	0.67	−0.43	−0.36	−0.50
**Apigenin glucoside**	0.17	0.24	−0.10	−0.38	0.18
**Apigenin**	0.16	0.44	−0.23	0.05	0.41
**Kaempferol pentoside iso 1**	0.31	0.61	−0.28	−0.07	0.15
**Kaempferol pentoside iso 2**	0.13	0.38	−0.35	−0.09	0.34
**Digalloyl HHDP iso 1**	0.26	0.68	−0.38	−0.07	−0.09
**Digalloyl HHDP iso 2**	0.19	0.75	−0.52	0.13	−0.27
**Digalloyl HHDP iso 3**	−0.36	−0.16	−0.40	−0.50	−0.22

**Casuariin**	0.08	0.46	−0.39	−0.30	−0.13
**Pedunculagin iso 1**	−0.24	0.36	−0.59	−0.10	−0.29
**Pedunculagin iso 2**	−0.27	0.37	−0.67	0.04	−0.31
**Galloylhexoside iso 1**	0.07	0.19	−0.07	0.20	0.57
**Galloylhexoside iso 2**	−0.20	0.17	−0.78	0.07	−0.11
**Galloylhexoside iso 3**	−0.31	−0.08	−0.61	0.07	−0.22
**Gallocatechin**	−0.16	0.12	−0.08	0.36	0.34
**Ellagic acid**	−0.32	−0.44	−0.14	−0.14	−0.18
